# Single‐Nucleus Atlas of Spinal Sarcopenia Patients Reveals Disruption of Quiescent and Regenerative Satellite Cell Niches in Fatty‐Infiltrated Muscle

**DOI:** 10.1002/advs.77648

**Published:** 2026-10-04

**Authors:** Wenkai Wu, Renxian Wang, Dingding Wang, Quanzhong Ren, Ling Wang, Fangfang Duan, Guihe Yang, Ruixuan Yu, Yi Wang, Yingyu Zhang, Zetong Zang, Chuqi Li, Weijia Liu, Wenshuang Zhang, Yi Yuan, Zitong Cheng, Xiaoguang Cheng, Junbo Liang, Yajun Liu

**Affiliations:** ^1^ Department of Spine Surgery Beijing Jishuitan Hospital National Centre for Orthopaedics Capital Medical University Beijing China; ^2^ JST sarcopenia Research Centre Beijing Research Institute of Traumatology and Orthopaedics National Center for Orthopaedics Beijing Jishuitan Hospital Capital Medical University Beijing China; ^3^ Department of Radiology Beijing Jishuitan Hospital National Centre for Orthopaedics Capital Medical University Beijing China; ^4^ Clinical Epidemiology Research Center Beijing Jishuitan Hospital National Centre for Orthopaedics Capital Medical University Beijing China; ^5^ Union Medical University Center for Bioinformatics Department of Biochemistry and Molecular Biology Institute of Basic Medical Sciences Chinese Academy of Medical Sciences School of Basic Medicine Peking Union Medical College Beijing China; ^6^ Biomedical Sciences College Shandong Medicinal Biotechnology Centre Shandong First Medical University & Shandong Academy of Medical Sciences Jinan China

**Keywords:** adipokine, fatty infiltration, microenvironment, sequencing, spinal sarcopenia

## Abstract

Severe muscle fat infiltration is a contributing factor to muscle loss, particularly in the spinal sarcopenia population. However, the underlying mechanism largely remains elusive. Here, we identified the intramuscular fat fraction (IMFF), rather than perimuscular fat fraction, as an independent determinant of trunk muscle strength. Individuals with higher IMFF (10‐15% and >15%) exhibited significantly lower paraspinal muscle mass and strength compared to those with lower IMFF (<5%). Using single‐nucleus RNA sequencing, we established the first cellular atlas of fat‐infiltrated paraspinal muscle, revealing profound remodeling of muscle architecture, extracellular matrix organization, and satellite cell niches. Among the adipose‐derived factors, ANGPTL4 emerged as a key paracrine suppressor of myogenesis, acting through upregulation of syndecan‐2 (SDC2) in satellite cells. Inhibition of SDC2 partially rescued ANGPTL4‐induced myogenic impairment in C2C12 cells. Furthermore, we identified a GPC3^+^ fibro/adipogenic progenitor (FAP) subpopulation with enhanced lipogenic potential, characterized by high expression of GPC3, PPARG, ADIPOQ, and FABP4, representing a transition from MME^high^ GPC3^low^ FAPs into MME^low^GPC3^high^ adipogenic FAPs. Collectively, our findings delineate the cellular landscape and signaling interactions that underlie pathological fat infiltration in spinal muscles, providing mechanistic insight and potential therapeutic targets for spinal sarcopenia.

## Graphical abstract

Mechanisms of Fatty Infiltration and Muscle Degeneration. Clinical metadata identified intramuscular fat accumulation as an independent driver of muscle mass decline. Single‐nucleus RNA sequencing of spinal sarcopenia muscle revealed adipogenic reprogramming of fibro‐adipogenic progenitors, satellite‐cell niche disruption, and elevated levels of the anti‐myogenic adipokine ANGPTL4—mechanistic alterations that synergistically accelerate muscle degeneration within fat‐infiltrated regions.

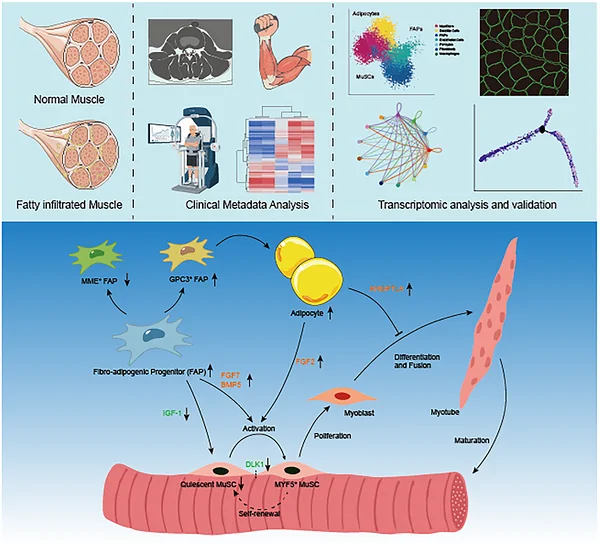

**Abbreviations**
ANGPTL4: angiopoietin‐like 4
FAP: fibro‐adipogenic progenitor
IMF: intramuscular fat
IMFF: intramuscular fat fraction
MFI: muscle fat infiltration
MSC: mesenchymal stem cell
MuSC: muscle satellite cell
PMFF: perimuscular fat fraction
PSMI: paraspinal muscle index
SDC2: syndecan‐2
SMI: skeletal muscle index

## 1 Introduction

Degenerative spine diseases (DSDs) constitute a growing global health challenge, ranking among the leading causes of disability and socioeconomic burden across populations [1]. Despite their high prevalence, particularly in ageing societies where rates exceed 25% [2], the biological processes linking structural degeneration to functional decline remain poorly understood. Emerging evidence highlights paraspinal muscle fatty infiltration (MFI) as a critical yet underexplored hallmark of DSDs, consistently associated with greater symptom severity, impaired function, and adverse surgical outcomes [3, 4, 5, 6]. Unlike other skeletal muscles, paraspinal muscles undergo disproportionate fatty degeneration—termed spinal sarcopenia [7, 8, 9]—but the mechanisms driving intramuscular fat accumulation and its contribution to muscle loss remain elusive. Addressing this gap is essential to understand how MFI undermines spinal health and recovery.

The primary driver of IMF accumulation is the aberrant lipogenic differentiation of multipotent progenitor cells under pathological conditions. Early studies highlighted the potential flexibility in the source of IMF, with both myogenic (MuSCs, Myf5^+^ MSCs) and non‐myogenic progenitor cells (fibro/adipogenic progenitors (FAPs), fibroblasts, endothelial cells (ECs), pericytes, myeloid‐derived cells, etc.) demonstrating lipogenic capacity in vitro, among which FAPs are regarded as the primary contributor to IMF [10, 11].Single‐cell/single‐nucleus sequencing (scRNAseq/snRNAseq) and bioinformatics tools have provided valuable insights into the cellular composition and pathological changes in MFI. Several studies have reported single‐cell profiles of fat‐infiltrated muscles mainly in animals, identifying FAP subpopulations with strong lipogenic capacity in vitro [12, 13, 14, 15]. However, the study of human muscle pathology is constrained by factors such as donor representation, variability between muscle groups, and challenges related to the accessibility and quality of muscle samples. Current research has primarily focused on aging‐related alterations in human muscle, with few studies investigating cellular compositional changes related to muscle fat infiltration within a specific muscle group [16, 17]. Three studies have reported possible adipogenic FAP subpopulations (MME^+^FAPs, DLK1^+^FAPs and VCAM^+^FAPs) in hip muscle, rotator cuff injured and chronic limb‐threatening ischemia (CLTI) muscles [12, 18, 19]. Curiously, both adipogenic FAPs were reduced in fat‐infiltrated muscles, a phenomenon that cannot be fully explained by the depletion caused by lipogenic differentiation. A key influencing factor may be the degree of pathological progression of MFI in the muscle samples. To date, no studies have identified a lipogenic intermediate‐state FAP subpopulation in human muscle, meaning that specific markers for lipogenic FAPs remain unidentified. Therefore, analyzing and cytomapping representative fat‐infiltrated muscle samples using single‐cell techniques is essential for understanding the pathogenesis of MFI.

How intramuscular fat (IMF) drives muscle loss remains unclear. Current models suggest that IMF creates a lipotoxic milieu, where saturated fatty acids such as palmitate promote insulin resistance and protein degradation, while inflammatory cytokines such as TNF‐α suppress myogenesis [20, 21]. Yet these mechanisms are largely inferred from in vitro studies, and in vivo evidence is lacking. Mitochondrial dysfunction and impaired mitochondrial biogenesis are direct contributors to sarcopenia. It is postulated that obesity‐induced chronic inflammation and reactive oxygen species may promote sarcopenia by triggering mitochondrial dysfunction [22]. The recent observation that adipokines like resistin exert age‐dependent effects on myogenesis further highlights an aged muscle–fat interaction underlying sarcopenia with fatty infiltration [23]. These gaps motivated us to dissect how IMF accumulation contributes to muscle wasting in MFI muscle samples.

To address this, we integrated clinical phenotyping, MRI‐based quantification, and functional assessments of paraspinal muscle in a clinical cohort of elderly inpatients with varying degrees of muscle fat infiltration. Surgical biopsies enabled multi‐modal analysis, combining histopathology, bulk transcriptomics, and snRNA‐seq to delineate the cellular and molecular landscape of spinal sarcopenia. Our findings uncover novel regulators of MFI‐associated muscle degeneration, offering potential therapeutic insights for a condition with profound implications for trunk disability and deformity.

## 2 Results

### 2.1 Correlation of Intramuscular Fat with Paraspinal Muscle Mass and Muscle Strength

The final analysis included 80 male and 81 female older patients (age ≥60 years) with lumbar spine MRI imaging and trunk muscle isokinetic strength data. Furthermore, from 98 patients (52 males and 46 females) in this cohort, the fat‐infiltrated muscle samples were analyzed by bulk RNA‐seq, and from 13 patients (7 females and 6 males), the samples were analyzed for single‐nucleus profiling (snRNA‐seq) (Figure 1A,B).

**FIGURE 1:**
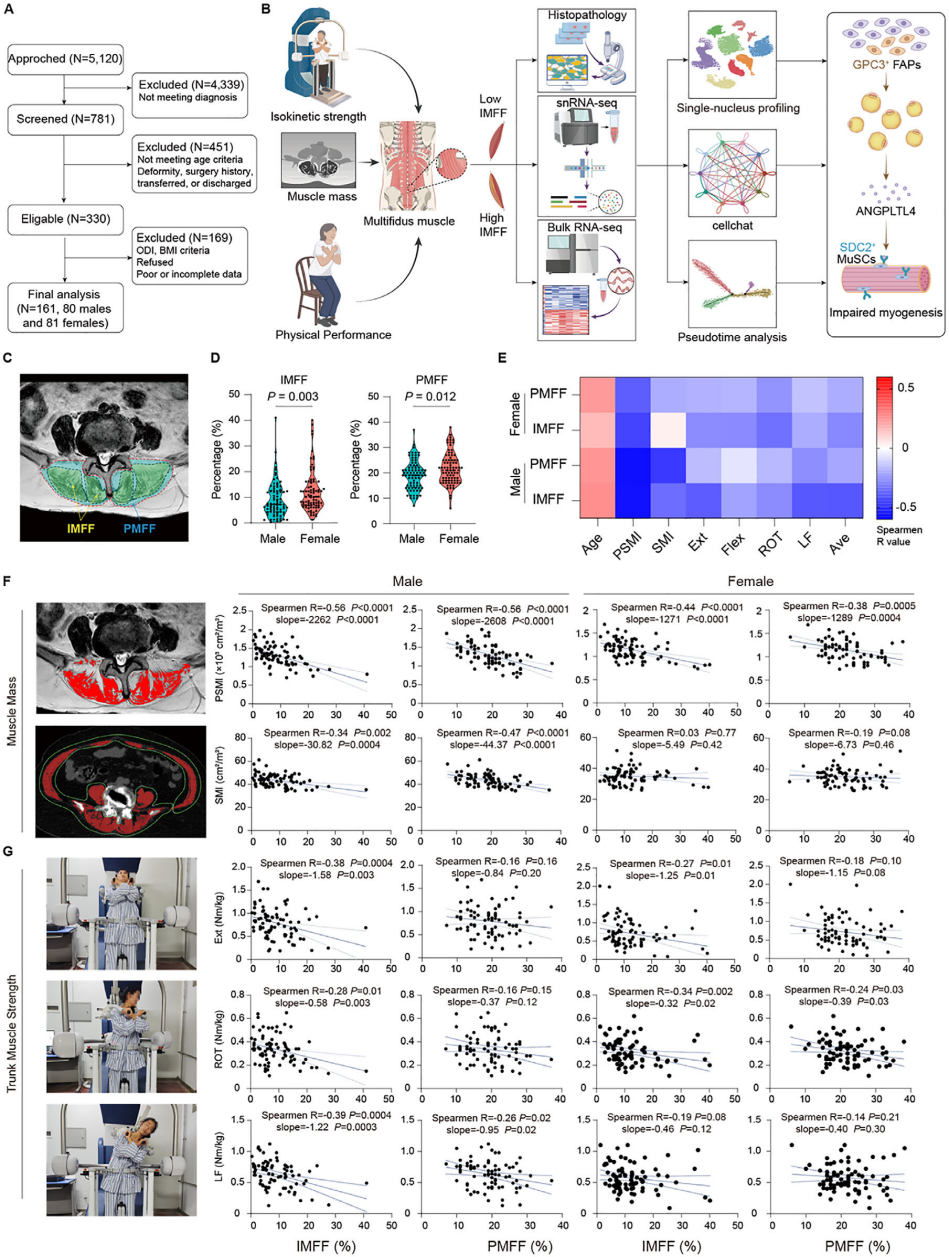
Intramuscular but not perimuscular fat fraction correlated significantly with isokinetic paraspinal muscle strength. A). CONSORT flowchart showing participant inclusion and exclusion in this study. A total of 80 males and 81 females were enrolled for the following statistical analysis. B). Schematic overview of the study design and main research objectives. C). Representative MRI image illustrating fat infiltration in the paraspinal muscles. The light blue area represents perimuscular fat, and the yellow area represents intramuscular fat. D). Comparison of intramuscular (IMFF) and perimuscular fat fraction (PMFF) between females and males. *P* value: Mann‐Whitney *U* test. E). Heatmap showing Spearman correlation coefficients between IMFF or PMFF and normalized paraspinal muscle area (named paraspinal muscle index, PSMI) and normalized isokinetic muscle strength in males and females. Strength metrics include extension (Ext), flexion (Flex), rotation (ROT), lateral flexion (LF), and average paraspinal muscle strength (Ave). Details can be found in Figure S1H.F,G). Scatter plots and simple linear regression analyses illustrating associations between IMFF or PMFF and muscle mass (PSMI and SMI), as well as normalized trunk muscle strength (Ext, ROT, and LF).

MRI analysis revealed significant sex differences in intramuscular and perimuscular fat fractions (IMFF and PMFF), with females exhibiting significantly higher values for both compartments (for IMFF, Mann‐Whitney *U* = 2355, *P* = 0.003; for PMFF, *U* = 2504, *P* = 0.012; Figure 1C,D). Although IMFF and PMFF were moderately correlated (Spearman *r* = 0.51 and 0.46 in males and females, *P<*0.001), their age‐related trajectories diverged dynamics differed. IMFF increased more steeply with age (slope = 0.38 and 0.41 in males and females, *P<*0.05) compared to PMFF (slope = 0.25 and 0.24 in males and females, *P<*0.05 and *P =* 0.09) (Figure S1A,B). Spearman and univariate linear regression analyses revealed that both IMFF and PMFF were negatively associated with muscle mass (normalized paraspinal muscle index (PSMI) and cross‐sectional skeletal muscle index (SMI), cm^2^/m^2^) (Spearman r = ‐0.34 to ‐0.56, *P*<0.01), with the exception of SMI in females (*P*>0.05). However, compared to the PMFF, IMFF demonstrated stronger negative correlations with trunk muscle strengths (normalized kinetic extension (Ext), rotation (ROT) and lateral flexion (LF) strength, Nm/kg) (Spearmen r = ‐0.27 to ‐0.39, *P*<0.05; slope = ‐0.32 to ‐1.58, *P*<0.05), except for LF in females (*P*>0.05) (Figure 1E–G, Figure S1H). Multiple linear regression further confirmed that IMFF, but not PMFF, was an independent determinant of trunk extension (*β =* ‐0.809, *P<*0.05), rotation (*β =* ‐0.312, *P<*0.05), and lateral flexion (*β =* ‐0.448, *P<*0.05) strength after adjustment for age, sex, BMI, ODI, and SMI (Table S1). Taken together, these findings highlight the significant detrimental effects of IMFF on both muscle mass and strength, whereas PMFF is only associated with muscle mass. Consequently, IMFF was used as a grouping variable for subsequent analyses.

Unfortunately, no significant correlations were observed between IMFF or PMFF and systemic indicators of sarcopenia, including GS, standing test (ST), step speed (Spd), and the short physical performance battery (SPPB) (Figure S1C–H). These findings suggest that the impact of IMFF on paraspinal muscles is localized, indicating that spinal sarcopenia, defined by paraspinal MFI, is mechanistically distinct from generalized sarcopenia.

### 2.2 High Intramuscular Fat Levels Impact Paraspinal Muscle Mass and Strength

Restricted cubic spline (RCS) regression adjusted for age, sex, and ODI revealed non‐linear associations between IMFF and trunk extension and lateral flexion strength (*P*<0.001 and *P* = 0.005). IMFF showed threshold effects for extension (inflection point =  0.07, *P* for likelihood < 0.001) and lateral flexion (inflection point =  0.06, *P* for likelihood  =  0.005), where strength declined steeply below the thresholds and plateaued thereafter (Figure 2A, Figure S2A,B and Table S2). TMFF exhibited a similar pattern with an inflection at 0.23 (*P* for non‐linearity  =  0.018; *P* for likelihood  =  0.034), whereas PMFF showed no adverse association, displaying a positive correlation with extension strength only above 0.32 (*P* for non‐linearity  =  0.036; *P* for likelihood  =  0.031). These results indicate an early, steep loss of extensor strength with rising IMFF and TMFF but not PMFF, followed by a plateau once IMFF exceeds ∼7% or TMFF exceeds ∼23%.

**FIGURE 2:**
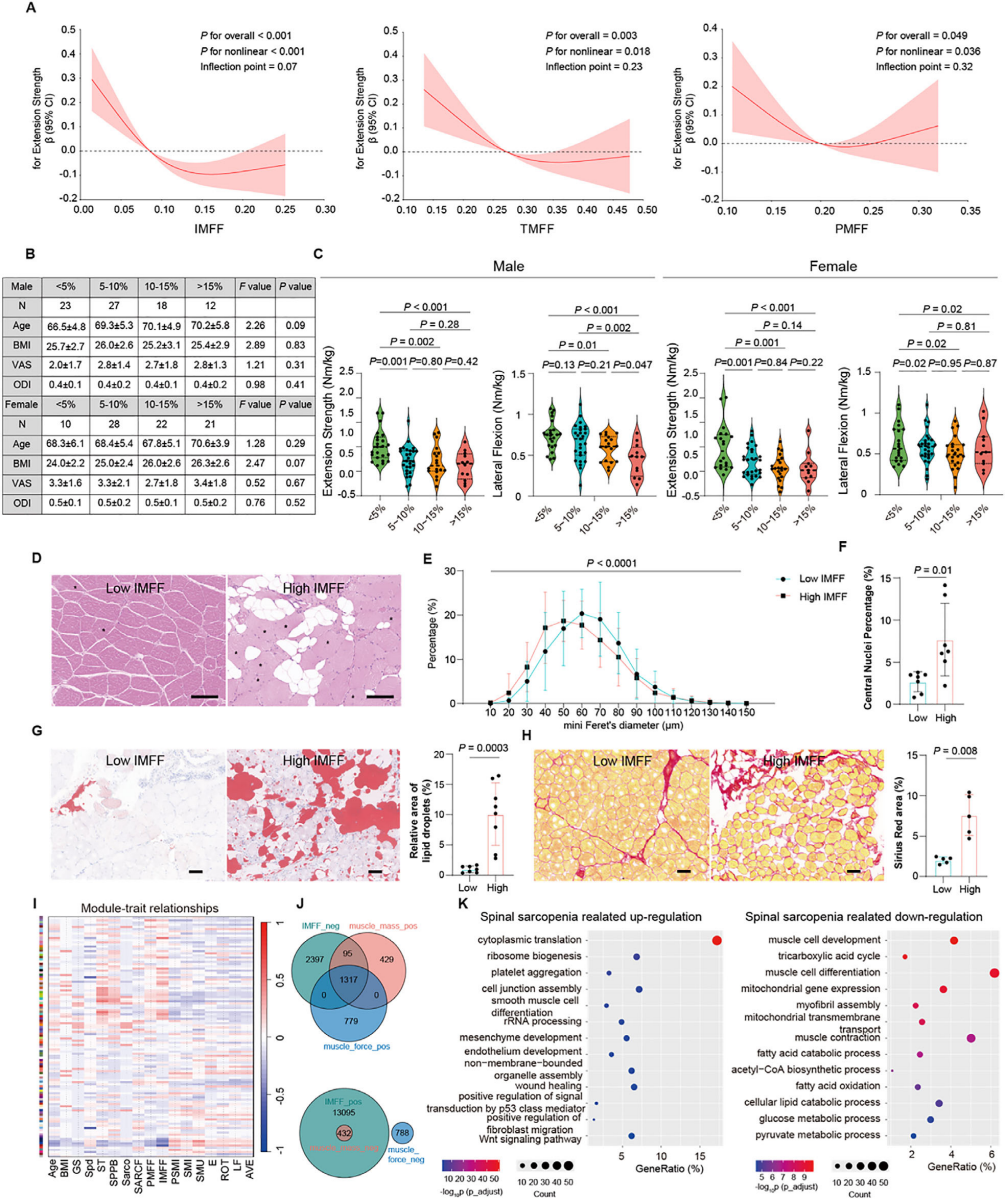
Inflection point of muscle fat infiltration and corresponding functional, pathological, and transcriptomic characteristics. A). Restricted cubic spline (RCS) models depicting the non‐linear associations of intramuscular (IMFF), perimuscular (PMFF), and total muscle fat fraction (TMFF) with standardized trunk extension strength, adjusted for sex, age, and ODI. B). Baseline characteristics of participants in the four IMFF groups (IMFF <5%, 5–10%, 10–15%, and ≥15%). *P* value: one‐way analysis of variance (ANOVA). C). Comparison of normalized trunk extension and lateral flexion strength among IMFF groups by one‐way ANOVA with post hoc Tukey's test. Details can be found in Table S5. D‐F). Representative HE staining of low IMFF (<5%) and high IMFF (>15%) muscle samples, with quantification of myofiber size (measured as minimum Feret's diameter; Mann‐Whitney *U* test) and centrally nucleated fibers (indicated by asterisks; Mann‐Whitney *U* test). Scale bar, 100 µm. G, H). Representative Oil Red O and Picrosirius Red staining of low and high IMFF muscle samples. Scale bar, 100 µm. *P* value: Mann‐Whitney *U* test. I, J). Weighted gene co‐expression network analysis (WGCNA) linking clinical phenotypes with transcriptomic modules. In total, 1317 down‐regulated and 432 up‐regulated genes were identified as associated with spinal sarcopenia. K). Gene Ontology (GO) enrichment analysis of the up‐ and down‐regulated genes associated with spinal sarcopenia.

Given the skewed distribution of IMFF, patients were categorized into four groups (<5%, ≥5–10%, 10–15%, and ≥15%) based on quartiles (Figure 2B). Intergroup comparisons showed markedly lower paraspinal muscle mass (PSMI, SMI, etc.) and normalized trunk muscle strength (Ext, LF, ROT, etc.) in individuals with IMFF >10% or 15% than in those with IMFF <5%. (Fig 2C, Figure S2C–E and Table S5) No significant differences (*P*>0.05) were identified in age, BMI, VAS (back pain), or Oswestry Disability Index (ODI) across groups (Figure 2B). These findings suggest that muscle fat infiltration may contribute to muscle deterioration in a cumulative manner, with significant reductions in muscle mass and strength observed in individuals with IMFF exceeding 10% and 15% compared to those with lower IMFF levels.

We then performed a pathological analysis of representative multifidus muscle samples with high (>15%) and low (<5%) IMFF levels and observed more pronounced muscle degeneration in the high IMFF group. First, the reduction in fiber size was evident, with a decrease in the proportion of medium‐ and large‐sized muscle fibers and an increase in small‐sized fibers (*U* = 24464026, *P*<0.001) (Figure 2D,E). Second, there was a significant increase in centrally nucleated muscle fibers, a hallmark of muscle injury or myopathy (*U* = 5, *P* = 0.01) (Figure 2F). Furthermore, intermyofibrillar adipose and fibrotic tissue became more prominent in these muscles as shown in Oil Red O (*U* = 0, *P* = 0.0003) and Picrosirius Red (PSR) staining (*U* = 0, *P* = 0.008) (Figure 2G,H). Meanwhile, we examined 98 samples from this cohort to establish the relationship between the transcriptional profiles and clinical phenotypes using weighted correlation network analysis (WGCNA) (Figure 2I,J). There were 432 upregulations and 1317 downregulations identified that seemed to be closely associated with fat infiltration, low muscle mass, and low muscle strength. As shown in the Gene Ontology (GO) enrichment analysis, the processes including cytoplasmic translation, endothelium development, and mesenchyme development were enhanced, whereas the activities of muscle cell development, muscle contraction, the tricarboxylic acid cycle, fatty acid catabolism, and mitochondria were diminished (Figure 2K). Therefore, the muscle with high IMFF exhibited significant muscle atrophy, mesenchymal remodeling, and dysregulation of lipid metabolism, all of which may contribute to the development of spinal sarcopenia.

### 2.3 The Microenvironment of Fat Infiltrated Paraspinal Muscles

We profiled the single‐nucleus transcriptomes of multifidus muscle biopsies from 7 low IMFF (IMFF<5%) and 6 high IMFF (>15%) donors undergoing spine surgery. A total of 112 502 nuclei were extracted, of which 112 154 nuclei were included for analysis (Figure 3A and Figure S3). Uniform manifold approximation and projection (UMAP) analysis delineated 12 major populations within low‐ and high‐fat‐infiltrated paraspinal muscles, including type I and II myofibers (MF‐I and MF‐II), muscle satellite cells (MuSCs), fibro/adipogenic progenitors (FAPs), fibroblast‐like cells, adipocytes, pericytes, smooth muscle cells (SMCs), endothelial cells (ECs), tenocytes, and immune lineages (myeloid and lymphoid cells) (Figure 3B). All 12 cell types were detected across samples, with cell proportion analysis revealing reduced MF‐I (two‐tailed unpaired t‐test, t(11) = 3.14, *P* = 0.009) and MuSC (t(11) = 2.79, *P* = 0.02) populations but increased adipocytes (t(11) = 3.29, *P* = 0.007), FAPs (t(11) = 1.56, *P* = 0.15), ECs (t(11) = 2.55, *P* = 0.03) and immune cells (mainly lymphoid, t(11) = 1.31, *P* = 0.22, and myeloid, t(11) = 1.50, *P* = 0.16) in high‐IMFF muscles. (Figure 3C, Figure S4) Immunofluorescence staining confirmed the decrease in MF‐I (MYH7^+^, *U* = 1, *P* = 0.009), MuSCs (PAX7^+^, *U* = 0, *P* = 0.03) and the increase in FAPs (PDGFRa^+^, *U* = 0, *P* = 0.02) and ECs (VWF^+^, *U* = 0, *P* = 0.03) in the high IMFF muscles (Figure 3D–F, and Figure S4). These findings were further validated by the flow cytometric analysis, with MuSCs decreased by 41.07% (Lin^−^CD34^−^CD82^+^CD56^+^, *U* = 31, *P* = 0.04) and FAPs increased by 65.82% (Lin^−^CD34^+^CD56^−^, *U* = 32, *P* = 0.04) (Figure 3G). Collectively, the high IMFF paraspinal muscle microenvironment is marked by a decrease in MuSCs and MF‐I in contrast to the increase in adipocytes, FAPs, ECs, and immune cells, likely representing a disruption in muscle homeostasis.

**FIGURE 3:**
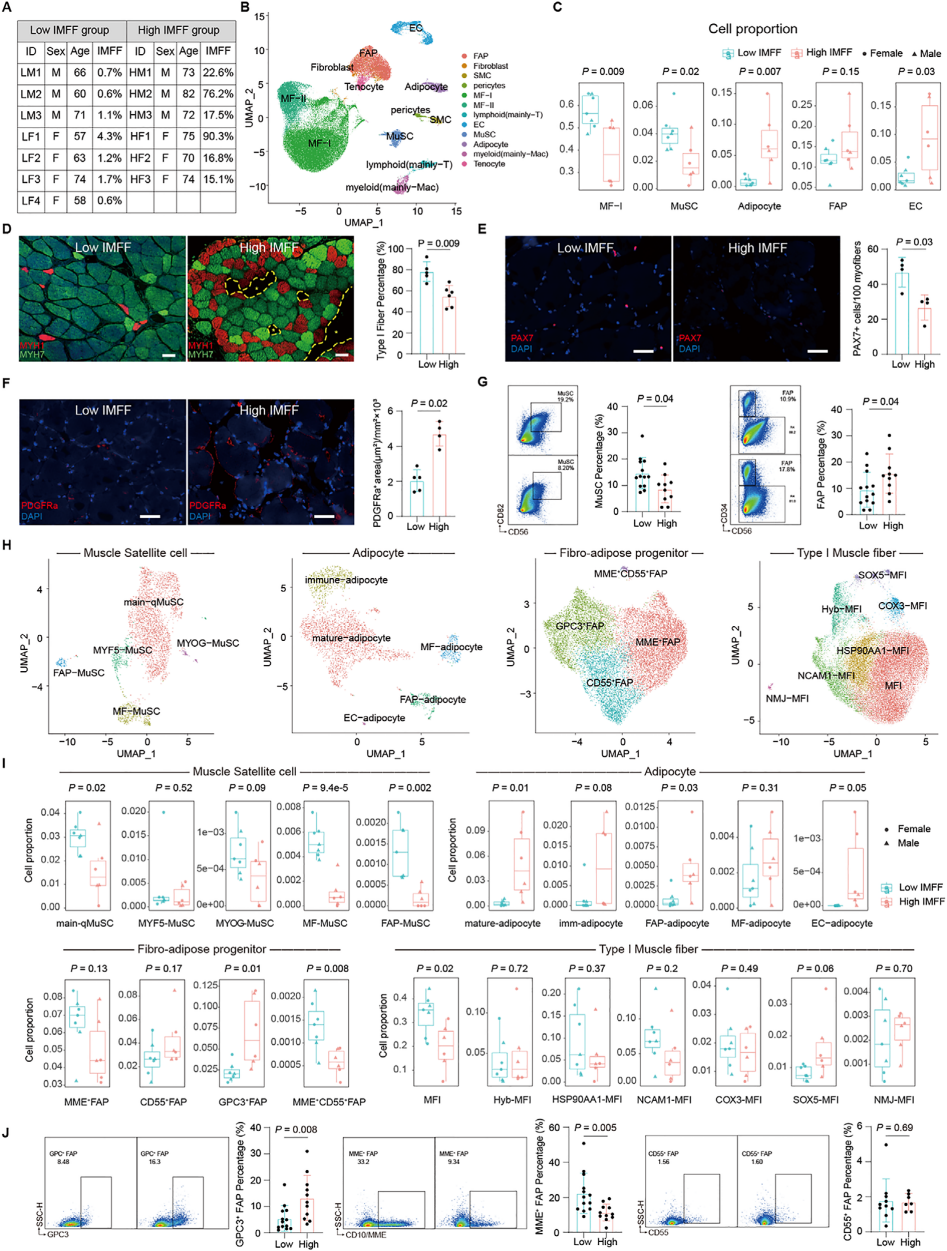
Single‐nucleus transcriptomic atlas of muscles with low and high intramuscular fat fraction (IMFF). A). Basic characteristics of donors and samples used for single‐nucleus sequencing (snRNA‐seq). B). UMAP visualization of the major muscle cell populations identified by snRNA‐seq. C). Quantification of the relative proportions of major cell populations in low and high IMFF muscle samples. D). Immunofluorescence staining and quantification of type I muscle fibers in low and high IMFF multifidus muscles (five low IMFF versus six high IMFF donors). Scale bar, 50 µm. Intramuscular adipocytes were noted with asterisks and yellow dashed circles. *P* value: two‐tailed unpaired t‐test. E, F). Immunofluorescence staining and quantification of PAX7^+^ cells and PDGFRa^+^ areas in muscle tissues. Scale bar, 50 µm. *P* value: two‐tailed unpaired t‐test. G). Flow cytometry analysis comparing the proportions of MuSCs and FAPs in multifidus muscle single‐cell suspensions from low IMFF (n = 13) and high IMFF (n = 10) groups. *P* value: two‐tailed unpaired t‐test. H). UMAP visualization of nuclear subpopulations within MuSCs, adipocytes, FAPs, and type I muscle fibers. I). Proportion analysis of nuclear subpopulations within MuSCs, adipocytes, FAPs, and type 1 muscle fibers between low and high IMFF groups. J). Flow cytometry analysis of FAP subset proportions in multifidus muscle single‐cell suspensions from low IMFF (n = 13) and high IMFF (n = 11) groups. *P* value: two‐tailed unpaired t‐test.

We then zoomed in on the MF‐I, MF‐II, MuSCs, adipocytes, FAPs, and EC populations to gain further insights into the microenvironment of high IMFF muscles (Figure 3H, Figure S5A–H). PAX7^+^ MuSCs were classified into five subclusters, including the main quiescent MuSCs (main‐qMuSCs, CALCR^+^), early‐activated MYF5‐MuSCs (MYF5^+^), late‐activated MYOG‐MuSCs (MYOG^+^), and two new subtypes, i.e., MF‐MuSCs (MYH7^+^) and FAP‐MuSCs (PDGFRa^+^, DCN^+^). MF‐MuSCs were enriched for myofiber markers MYH7, MYH1, and MYH2, suggesting a myogenic differentiation state more advanced than MYOG^+^ MuSCs [24]. FAP‐MuSCs expressed a high level of decorin (DCN), a known inhibitor of myostatin (MSTN) [25], suggesting that FAP‐MuSCs may play a role in counteracting muscle atrophy. Adipocytes were categorized into mature adipocytes (ADIPOQ^+^, PLIN1^+^), immune‐associated adipocytes (ANXA1^+^), in addition to three new adipocyte subpopulations, i.e., EC‐adipocytes (VWF^+^), FAP‐adipocytes (PDGFRa^+^), and MF‐adipocytes (MYH7^+^), which likely represent the subpopulations at different stages of differentiation. The nuclei of myofibers (MF‐I and MF‐II) were composed of several subgroups associated with main MFI (MYH7^+^) and MFII (MYH1^+^) fibers, hybrid fibers (MYH7^+^ and MYH1^+^), neuromuscular junction‐related fibers (NMJ‐MFI, MUSK^+^), and four subgroups with characteristic genes linked to proteostasis (HSP90AA1^+^), reinnervation (NCAM1^+^), energy metabolism (COX3^+^), and myogenesis (SOX5^+^) [26, 27, 28]. In addition, FAPs were categorized into four subtypes, i.e., GPC3^+^, MME^+^, CD55^+^, and MME^+^CD55^+^ subgroups, as previously reported [12], while arterial ECs (GJA5^+^), venous ECs (ACKR1^+^), capillary ECs (CA4^+^), lymph ECs (PROX1^+^) [29], and two new subtypes: FAP‐ECs (PDGFRa^+^), and MF‐ECs (MYH7^+^), collectively contributed to the EC compartment.

Furthermore, as shown in Figure 3I, the proportions of main‐qMuSCs (two‐tailed unpaired t‐test, t(11) = 2.78, *P* = 0.02), late‐myogenic MF‐MuSCs (t(11) = 5.96, *P* = 9.4e‐5), and anti‐atrophy FAP‐MuSCs (t(11) = 4.09, *P* = 0.002) were significantly reduced in the high‐IMFF group, suggesting that disruption of these MuSC subpopulations may contribute to impaired muscle regeneration. Although only the main MFI subgroups showed a significant decrease (t(11) = 2.75, *P* = 0.02), HSP90AA1^+^MFI and NCAM1^+^MFI also showed decreases, albeit not statistically significant, suggesting that impaired myofibrillar protein homeostasis and reinnervation may contribute to the decrease in paraspinal muscle mass [27, 28]. In contrast, the increase in myogenic‐related SOX5^+^ MFI fibers suggests an active myogenic process, potentially serving as a compensatory response to muscle loss [26]. Taken together, the reduction in main type 1 muscle fibers is accompanied by impaired proteostasis and decreased reinnervation of muscle fibers, which may be directly responsible for paraspinal muscle loss [28]. Meanwhile, all subtypes of adipocytes (mature‐adipocyte, immune‐adipocyte, FAP‐adipocyte, etc.) showed moderate increases, led by the mature subtype (t(11) = 2.97, *P* = 0.01). The FAP subgroups also varied in the high IMFF group, showing an increase in GPC3^+^FAPs (t(11) = 2.90, *P* = 0.01) and CD55^+^FAPs (t(11) = 1.46, *P* = 0.17), and a decrease in MME^+^FAPs (t(11) = 1.64, *P* = 0.13) and MME^+^CD55^+^FAPs (t(11) = 3.24, *P* = 0.008). This observation was largely in line with the flow cytometric analysis of fresh muscle samples (Figure 3J), in which GPC3^+^FAPs (Lin^−^CD34^+^CD56^−^GPC3^+^) increased by 2.42‐fold (*U* = 26.5, *P* = 0.008) while MME^+^FAPs (Lin^−^CD34^+^CD56^−^CD10^+^) (*U* = 24, *P* = 0.005) decreased by 0.51‐fold (Figure 3J). FAPs serve as pivotal regulators that both preserve muscle homeostasis and drive intramuscular adipogenesis. Here, we identified a marked reduction of MME^+^FAPs accompanied by a concomitant expansion of GPC3^+^FAPs in muscles with high IMFF, a shift that may underlie intramuscular fat accumulation and the breakdown of muscle homeostasis.

### 2.4 Fat Infiltration Disrupts MuSC Autonomous Signaling and Niche Integrity

To explore fat‐muscle interactions associated with muscle loss, we applied CellChat analysis to delineate intercellular communication networks within fat‐infiltrated muscles. Muscles with high IMFF exhibited globally enhanced signaling activity, particularly in pathways related to growth factors, cell–cell contact, matrix remodeling, immune modulation, and vascular and neural regeneration (Figure 4A,B). Signals targeting MuSCs were markedly strengthened and primarily involved pathways promoting activation and differentiation (NOTCH, FGF, BMP, PDGF, HGF) or fibrogenic remodeling (COLLAGEN, JAM). Notably, newly identified atrophy‐associated THBS1 and adipokine ANGPTL4 emerged as active inputs to MuSCs in high‐IMFF muscles (Figure 4C, Figure S6A–J).

**FIGURE 4:**
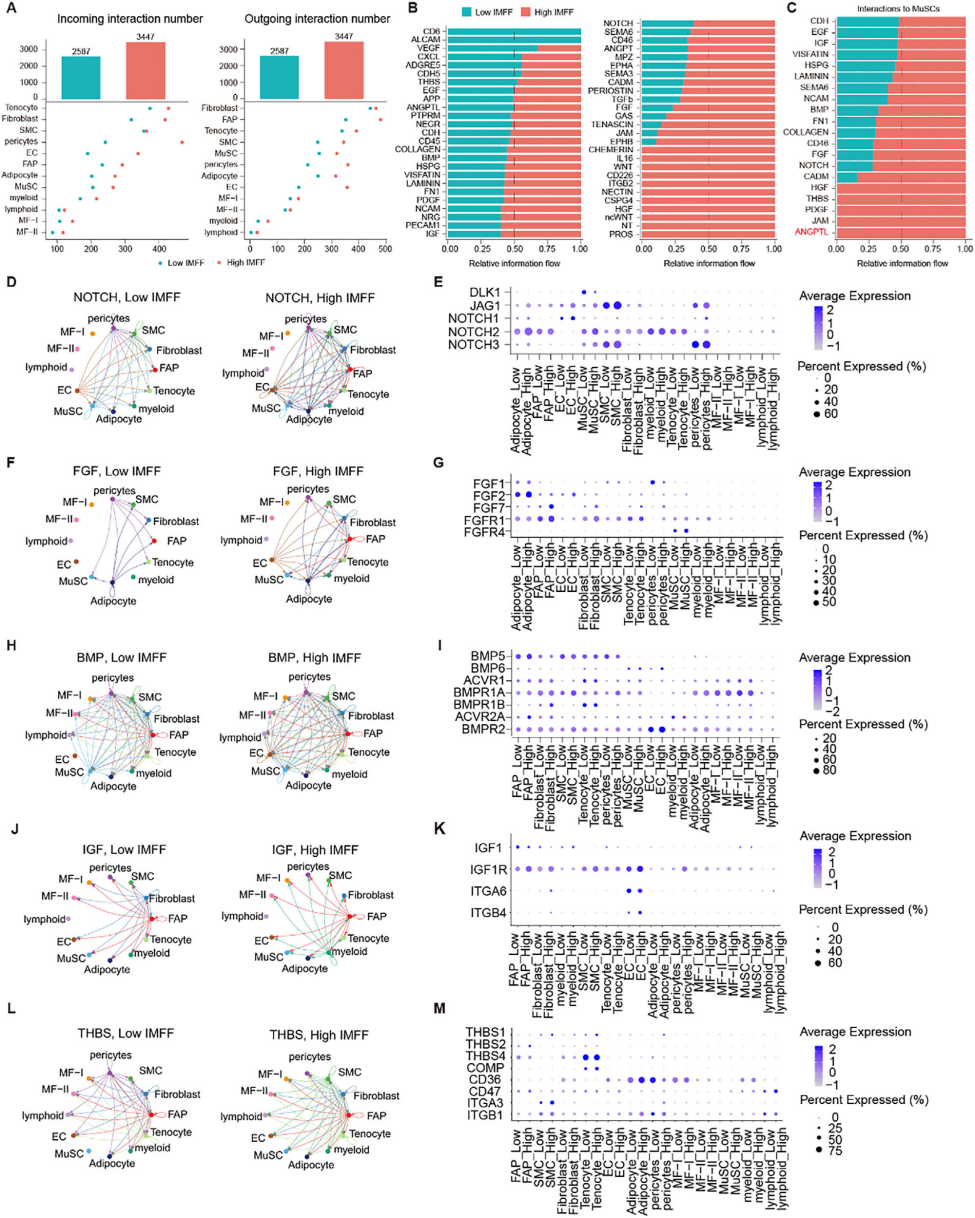
Altered cellular communication in fat‐infiltrated muscles. A). Number of predicted incoming and outgoing interactions for each cell type in the low (green) and high (red) IMFF groups. B). Relative information flow, represented by the summed interaction probabilities, for each signaling pathway in low and high IMFF muscles. C). Differences in the probability of incoming interactions (relative information flow) received by satellite cells between low IMFF (green) and high IMFF (red) groups. D‐M). Representative signaling networks in low and high IMFF muscles. Cell types are shown as nodes and their interactions as edges, with edge width proportional to interaction probability. The accompanying dot plots depict the expression of genes associated with each signaling pathway, where color intensity indicates average expression and dot size denotes the proportion of cells expressing each gene within a given cell type.

In low‐IMFF muscles, MuSCs maintained quiescence mainly through self‐regulatory DLK1‐NOTCH2/3 interactions, whereas in high‐IMFF muscles, DLK1 expression was markedly reduced (Figure 4D,E). Instead, MuSCs received intensified NOTCH inputs from surrounding FAPs, endothelial cells, pericytes, and adipocytes, driven by upregulation of JAG1 and NOTCH2/3, suggesting a disruption of the MuSC quiescent niche. Moreover, FAPs and adipocytes exerted additional influence via enhanced FGF2/7‐FGFR4 signaling, while FGF1 was downregulated in FAPs and pericytes (Figure 4F,G). FAP‐derived BMP5‐ACVR2A signaling was also increased, despite reduced IGF1 expression (Figure 4H–K). The myogenic inhibitor THBS1 was found to be overexpressed in pericytes, tendon cells, smooth muscle cells, and fibroblasts, and targeted MuSCs despite its overall low expression levels in high‐IMFF muscles (Figure 4L,M). Collectively, these alterations in intercellular communication indicate a breakdown of MuSC quiescent homeostasis and a shift in local paracrine signaling within high‐IMFF muscles, which may compromise MuSC self‐renewal and differentiation potential.

### 2.5 Adipocyte‐Derived ANGPTL4 Inhibited MuSC Myogenesis

ANGPTL4, as an adipokine, is known to inhibit lipoprotein lipase (LPL) and myogenesis [30]. In high‐IMFF muscles, we identified a newly emergent ANGPTL4 signaling axis from adipocytes to MuSCs. Given that ANGPTL4 expression was not elevated within individual adipocytes, this interaction is likely driven by adipocyte expansion and upregulation of the ANGPTL4 receptor Syndecan‐2 (SDC2) in MuSCs (Figure 5A,B, Figure S7A,B) [31]. Subcluster analysis further revealed that immune‐adipocytes exhibited higher ANGPTL4 expression, whereas all MuSC subpopulations showed increased SDC2 expression (Figure S7C,D).

**FIGURE 5:**
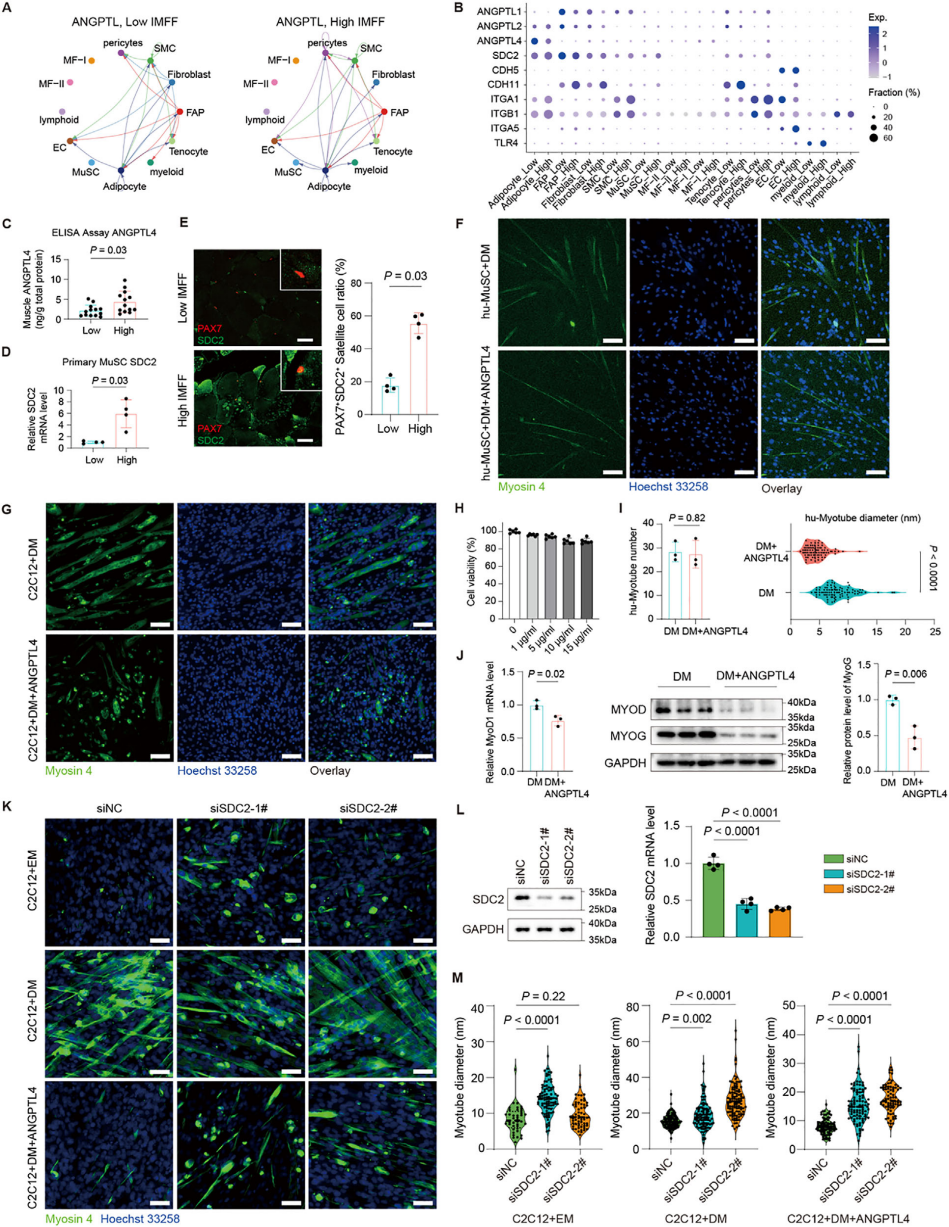
Adipocyte‐derived ANGPTL4 inhibited MuSC myogenesis. A). ANGPTL signaling network in low and high IMFF muscles. Cell types are represented by nodes and their interactions by edges, with edge width proportional to interaction probability. B). Expression profiles of genes associated with the ANGPTL signaling pathway in low and high IMFF muscles. The color scale indicates average gene expression, and the dot size indicates the percentage of cells expressing the gene within each cell type. C). Levels of ANGPTL4 in low and high IMFF muscle tissues measured by ELISA (n = 14). *P* value: two‐tailed unpaired t‐test. D). Fold change in SDC2 expression measured by qRT‐PCR in flow cytometrically sorted primary satellite cells from low and high IMFF multifidus muscles. *P* value: two‐tailed unpaired t‐test. E). Immunofluorescence staining of SDC2 and PAX7 in low and high IMFF muscles. The proportion of PAX7^+^SDC2^+^ satellite cells among total PAX7^+^ cells was compared using a two‐tailed unpaired t‐test. F). Representative immunofluorescence images showing myotube formation in primary human MuSCs (hu‐MuSC) cultured for 5 days in differentiation medium (DM) with or without ANGPTL4. Nuclei were stained with Hoechst 33258 (blue) and myosin heavy chain (MYH4) with a fluorescent antibody (green). Scale bar, 100 µm. G). Representative immunofluorescence images showing myotube formation in C2C12 myoblasts cultured for 5 days in DM or DM supplemented with ANGPTL4. H). Viability of C2C12 cells treated with different concentrations of ANGPTL4. I). Quantification of myotube number and diameter. *P* value: two‐tailed unpaired t‐test. J). Fold change in *Myod1* expression measured by qRT‐PCR in C2C12 myoblasts after 5 days of differentiation. *P* value: two‐tailed unpaired t‐test. Western blot analysis showing MYOD and MYOG expression in differentiated C2C12 cells cultured with or without ANGPTL4. K). Representative immunofluorescence images of myotubes after 5 days of culture in expansion medium (EM), DM, and DM+ANGPTL4 using C2C12 myoblasts pre‐transfected with siNC, siSDC2#1, or siSDC2#2. L). Western blot and qRT‐PCR analysis of SDC2 expression in C2C12 cells at 72 h after 24 h of siRNA pre‐transfection. *P* value: one‐way ANOVA with Dunnett multiple comparison. M). Comparison of myotube diameters among different groups. P value: Kruskal‐Wallis test with Dunn's multiple comparison.

Consistently, ELISA and qRT–PCR analyses showed elevated ANGPTL4 in high‐IMFF muscles (*U* = 52, *P* = 0.03) accompanied by increased SDC2 mRNA (*U* = 0, *P* = 0.03) expression in primary MuSCs derived from high‐IMFF patients (Figure 5C,D). Immunofluorescence confirmed an increased fraction of PAX7^+^SDC2^+^ MuSCs in these muscles (*U* = 0, P = 0.03, Figure 5E). Thus, the ANGPTL4‐SDC2 signaling was specifically enhanced in the high IMFF group, whose impact was further evaluated in vitro with both flow‐sorted MuSCs and C2C12 cells using Recombinant human ANGPTL4 protein (Figure 5F–G). CCK‐8 assays showed that ANGPTL4 (1–10 µg mL^−^^1^) had little effect on C2C12 viability (Figure 5H), but markedly reduced myotube nucleation (*U* = 752, *P*<0.0001) in MuSCs at 1 µg mL^−^^1^ despite unchanged myotube numbers (Figure 5I). Consistently, ANGPTL4 reduced the expression of downstream effectors in C2C12 cells, including Myod1 (–23.6%, two‐tailed unpaired *t*‐test, *t*(4) = 3.983, P = 0.02), and Myog (–52.6%, two‐tailed unpaired t‐test, *t*(4) = 5.321, *P* = 0.006) (Figure 5J). Meanwhile, we checked potential changes in C2C12 cells' myogenic differentiation upon depleting SDC2 (Figure 5K–M). The results showed that SDC2 knockdown promoted myogenic differentiation, comparable to the inhibitory effect of ANGPTL4 treatment. Moreover, the loss of SDC2 partially restored the myogenic differentiation of C2C12 cells that was inhibited by ANGPTL4. Thus, the adipocyte‐satellite cell crosstalk mediated by the ANGPTL4‐SDC2 pathway is critical for the suppression of myogenesis in the high IMFF muscle.

### 2.6 GPC3^+^FAPs Represent the Intermediate State of FAPs in Intramuscular Fat Formation

The presence of adipocyte subsets expressing FAP (PDGFRa, DCN, COL1A1), EC (VWF, PECAM1, ENG), and myofiber (MYH7, MYH1, MYH2) markers suggests that the subpopulations of these cell types may have lipogenic potential (Figure S5B). We next established the pseudotime trajectory analysis of various cell types identified in paraspinal muscles to explore the developmental relationship between muscle‐resident cells and adipocytes (Figure 6A, Figure S8A–N). Clustered at opposite ends of the trajectory were distinctive MuSCs and adipocyte subpopulations. There was no indication of adipogenic potential in MuSC subpopulations irrespective of the incorporation of myofibers into the analysis (Figure S8A–H). A distinct branch of FAP^+^ ECs exhibited some adipocyte features and may have acquired adipogenic potential. However, their proportion did not differ significantly between low and high IMFF muscles, possibly reflecting the generalized lipogenic capacity of ECs (Figure S8I–M). Similarly, a subset of GPC3^+^FAPs was clustered in the adipocyte branch (Figure 6B), and more importantly, was primarily distributed in the high IMFF muscle (Figure 6C). As shown in the differential gene enrichment (Figure 6D), the PPARγ signaling pathway (PPARG/LPL/CD36) and adipocytokine signaling pathway (LEPR/MAPK10/CD36) were both upregulated in this GPC3^+^FAP subset, highlighting an enhanced lipogenesis capacity. This finding was also backed by UMAP analysis, which identified a cluster of GPC3^+^FAPs with high expression of PPARG and FABP4, termed “adipogenic GPC3^+^FAPs” (Figure 6E). Based on these findings, we hypothesized that adipogenic GPC3^+^FAPs represent a FAP subpopulation undergoing lipogenic differentiation, positioning GPC3^+^FAPs as a critical FAP subpopulation driving adipogenesis.

**FIGURE 6:**
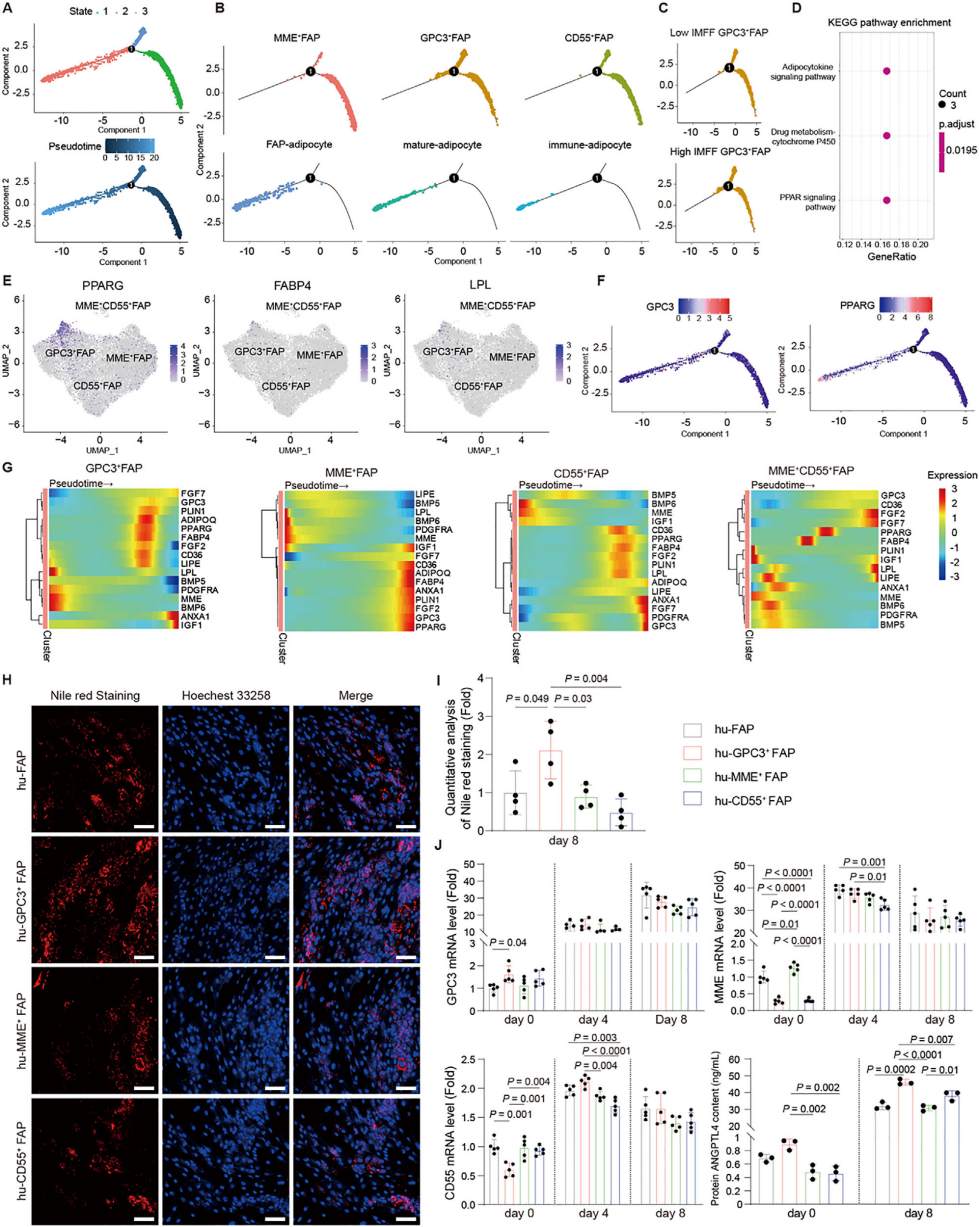
GPC3^+^FAPs represent the intermediate state of FAPs in intramuscular fat formation. A). Pseudo‐trajectory analysis identifying three distinct cellular states among FAPs and adipocytes. B). Distribution of FAP and adipocyte subpopulations among the inferred cell trajectories. C). Localization of GPC3^+^FAP along the trajectories in muscles with low and high IMFF. D). KEGG pathway enrichment analysis of genes differentially expressed between state 3 GPC3^+^FAPs and other FAPs. E). UMAP plots showing the expression of adipogenic markers across FAP subsets, with blue intensity indicating relative expression levels. F). Expression dynamics of GPC3 and PPARG along the cell trajectories. G). Pseudotime ordering of FAP subsets. The heatmap color gradient represents gene expression intensity across pseudotime progression. H). Nile red staining showing lipid accumulation in primary human FAPs (hu‐FAPs), hu‐GPC3^+^FAPs, hu‐MME^+^FAPs, and hu‐CD55^+^FAPs after 8 days of adipogenic incubation. I). Quantification of Nile red staining by ImageJ. Data are presented as fold change relative to unsorted FAPs (mean ± SEM; n = 3). *P* value: one‐way ANOVA with Tukey's multiple comparisons. J). Relative mRNA expression of GPC3, MME, and CD55 in FAPs during adipogenic differentiation (days 0, 4, and 8, n = 4), and ANGPTL4 levels in FAPs before (day 0, n = 3) and after (day 8, n = 3) adipogenic differentiation, measured by ELISA. *P* value: one‐way ANOVA with Tukey's multiple comparison.

In the trajectory, the GPC3^+^PPARG^+^ cells are positioned at the junction between the FAP and adipocyte arms (Figure 6F). Pseudotime‐based ranking of FAPs revealed a common sequential transition in gene expression: early‐stage FAPs exhibited high levels of MME and PDGFRa, while later‐stage cells displayed elevated expression of GPC3, FGF2/7, ANXA1, and lipogenic genes (PPARG, ADIPOQ, FABP4, and PLIN1) (Figure 6G). MME (CD10) and PDGFRa (CD140a) are well‐known mesenchymal stem cell (MSC) markers [32, 33, 34]; thus, the trajectory likely represented a FAPs differentiation transition from an MSC‐like state (high in MME and PDGFRa) to a developed adipogenic state (high in GPC3 and lipogenic genes) in paraspinal muscle fat infiltration.

We then validated the lipogenic capacity of FAPs flow‐sorted from human multifidus muscle. After 8 days of lipogenic induction, GPC3^+^FAPs generated significantly more lipid droplets than MME^+^FAPs and CD55^+^FAPs (one‐way ANOVA with post‐hoc Tukey's test, F(3, 12) = 6.98, *P* = 0.006; Figure 6H,I, and Table S6). Meanwhile, we monitored the expression of GPC3, MME, and CD55 in the course of lipogenesis (Figure 6J). Consistent with the pseudotime analysis, GPC3 expression progressively increased in all FAP subgroups, while MME and CD55 levels peaked at day 4 and subsequently declined (Figure 6J), which created a dynamic shift from MME^high^ GPC3^low^ to MME^low^GPC3^high^ during FAPs adipogenesis. Furthermore, we observed an increase in ANGPTL4 in the medium upon FAP adipogenic differentiation, especially from GPC3^+^FAPs (Figure 6I). Together, these findings confirmed strong adipogenic activities of GPC3^+^FAPs that underwent a transition from MME^high^ to GPC3^high^ states, accompanied by ANGPTL4 upregulation.

## 3 Discussion

We identify intramuscular fat fraction (IMFF), rather than perimuscular fat fraction (PMFF), as a key determinant of paraspinal muscle mass and trunk strength. IMFF showed a nonlinear relationship with muscle strength, with the negative association plateauing beyond 7%. Individuals with higher IMFF (10–15% and >15%) exhibited pronounced reductions in muscle mass and strength compared with those with low IMFF (<5%). Integrated histopathology and multi‐omic profiling revealed a distinct cellular architecture in fat‐infiltrated paraspinal muscle, marked by depletion of quiescent MuSCs and MME^+^ FAPs and expansion of adipocytes and GPC3^+^ FAPs. Altered intercellular communication indicated niche disruption and remodeling of muscle ECM and paracrine microenvironment. Mechanistically, we identified an ANGPTL4–SDC2 axis as a negative regulator of MuSC myogenesis in high‐IMFF muscles. Pseudotime analysis further highlighted GPC3^+^ FAPs as the main drivers of intramuscular adipogenesis, which likely requires a transition from MME^high^GPC3^low^ to MME^low^GPC3^high^. Together, these findings provide a cellular and molecular framework for pathological fat infiltration in spinal sarcopenia and uncover a previously unrecognized signaling mechanism linking adipocyte‐MuSC crosstalk to muscle degeneration.

Myosteatosis is associated with a variety of factors including aging, gender, obesity, and metabolic diseases, and is manifested by the accumulation of perimuscular and intramuscular fat [35, 36]. However, it remains unclear as to whether intramuscular and perimuscular fat have different impacts on muscle. Our study highlights that IMFF, rather than PMFF, is significantly associated with loss of paraspinal muscle mass and strength. Multiple linear regression showed that IMFF was an independent factor leading to decreased muscle strength and muscle loss, while the increase in PMFF was probably due to fat accumulation after muscle atrophy. This prompted us to investigate how intramuscular fat infiltration influences muscle fibers and resident cells through contact‐dependent and paracrine interactions.

Previous studies have linked MFI to reduced muscle mass and functional decline [11, 37]. However, the extent to which fat infiltration drives muscle degeneration remains unclear. Additionally, aging muscles appear to be more susceptible to the effects of anti‐myogenesis adipokines [23]. Here, we provide the first quantitative evidence defining the functional implications and threshold of intramuscular fat accumulation in the paraspinal muscles of older adults. Using RCS modeling, we identified a non‐linear association between IMFF and muscle strength, with a critical inflection at ∼7% (Figure 2A). Individuals with pronounced paraspinal fatty infiltration (IMFF exceeding 10% or 15%) exhibited markedly lower muscle mass and strength, suggesting a threshold beyond which muscle function deteriorates sharply (Figure 2C). These results underscore the need for standardized diagnostic criteria for spinal sarcopenia. Furthermore, the histological analyses of high IMFF muscles (>10%) revealed characteristic pathological changes, including smaller myofibers, increased central nuclei, and elevated inter‐myofiber fat and fibrosis (Figure 2D–H). WGCNA further connected reduced paraspinal muscle strength and muscle loss with a distinct transcriptional signature characterized by suppressed myogenic, energy, and lipid metabolism, accompanied by activation of tissue regeneration and mesenchymal remodeling (Figure 2I–K). Together, these findings provide an integrated view of the functional, structural, and molecular alterations underlying spinal sarcopenia and highlight key pathways warranting mechanistic investigation.

Generalized sarcopenia is typically marked by a significant decrease in type II muscle fibers [38]. In contrast, we observed a substantial reduction in type I muscle fibers in the fat‐infiltrated paraspinal muscle. This discrepancy may first be attributed to the nature of different muscle groups, as limb muscles predominantly contain type II fibers whereas paraspinal muscles are richer in type I fibers [39, 40]. In addition, MFI‐associated paraspinal sarcopenia is characterized by the loss of proteostasis and reduced reinnervation in type 1 muscle fibers, which may directly contribute to the development of paraspinal sarcopenia [28]. From the metabolic perspective, type I muscle fibers rely more on fatty acid oxidation for energy. The dysregulation of related metabolic pathways could impair energy homeostasis and muscle regeneration, leading to the decline in type I fibers, a process that may be largely driven by intramuscular fat infiltration (Figure 2I–K) [41]. This also contributes to the understanding of the aforementioned correlation between changes in fatty acid metabolism and muscle fiber composition in the course of paraspinal muscle fat infiltration.

IMF accumulation primarily arises from the abnormal adipogenesis of FAPs. Previous sc/snRNAseq studies have characterized the heterogeneity of FAPs in fat‐infiltrated muscles, identifying human MME^+^FAPs in hip muscles and DLK1^+^FAPs in rotator cuff muscles with higher lipogenic capacity in vitro [12, 18]. However, no studies have reported adipogenic intermediate‐state FAPs in human muscle, which may be attributed to the low abundance of adipogenic FAPs due to insufficient fat infiltration in the muscle. The present study, focusing on paraspinal muscles with severe MFI, reveals cellular composition changes and identifies a GPC3^+^FAP subpopulation that expresses the mature fat markers, providing crucial insights into FAP lipogenesis. First, we observed a specific increase in GPC3^+^FAPs in contrast to the decrease in MME^+^FAPs, which likely accounts for the FAP‐mediated lipogenesis that is among the primary sources of intramuscular fat accumulation [10]. Second, we observed IMF expansion‐related neovascularization and fibrosis. The dynamics of ECs were primarily driven by an increase in arterial and venous ECs, whereas capillary ECs remained unaltered. Of note, the observed increase in fibrosis in fat‐infiltrated muscle was consistent with a rise in CD55^+^FAPs (Figure 3I), a subpopulation identified as fibrogenic [18, 42]. Third, we identified diverse adipocyte subpopulations expressing respective FAP, EC, and MuSC markers, which likely reflect a collection of adipogenic intermediate states. Most importantly, we identified, for the first time, a cluster of GPC3^+^ FAPs aligned along the adipocyte trajectory and expressing mature adipocyte markers (PPARG, FABP4, LPL, etc.), predominantly from the high IMFF group, which may serve as a direct source of intramuscular fat. The relationship among FAP subgroups was deconvoluted in the pseudotime trajectory constructed, which was initiated by MME^+^PDGFRa^+^ FAPs and evolved into GPC3^+^FAPs which showed increased expression of adipogenic markers (PPARG, FABP4, ADIPOQ, etc.) (Figure 6G). Fitzgerald et al. and Garcia et al. reported that MME^+^FAPs and DLK1^+^FAPs exhibited greater adipogenic capacity but reduced presence in fat‐infiltrated muscle due to differentiation [12, 18]. Consistent with this, we observed a marked decline in MME^+^ FAPs alongside a prominent expansion of a GPC3^+^ subset that dominated the expanded FAP pool in fat‐infiltrated muscle. Pseudotime analysis identified an adipogenic intermediate subset within GPC3^+^FAPs and revealed a dynamic transcriptional trajectory from an MME^high^GPC3^low^ to GPC3^high^MME^low^ state, suggesting that GPC3^+^ FAPs represent a more advanced adipogenic stage. Functionally, GPC3^+^ FAPs isolated from high IMFF muscles displayed stronger adipogenic competence than MME^+^ FAPs, implying that the loss of MME^+^ cells largely reflects their transition toward the GPC3^+^ adipogenic fate. This phenotypic continuum provides a mechanistic explanation for the selective depletion of MME^+^ FAPs observed in high‐IMFF muscle [12]. Notably, the consistent increase in GPC3^+^ FAPs and the reduction in MME^+^ FAPs observed in both highly fat‐infiltrated gluteus minimus and multifidus muscles suggest that key aspects of FAP remodeling may be conserved across anatomically distinct skeletal muscles undergoing pathological fatty infiltration.

Intramuscular fat infiltration profoundly remodels the MuSC niche, disrupting muscle architecture and intercellular communication. Among the affected pathways, NOTCH signaling emerges as a pivotal regulator of MuSC fate, maintaining the balance between quiescence and activation through its key modulators JAG1 and DLK1 [43, 44]. The ligand Jagged1, absent in quiescent MuSCs, is rapidly induced upon activation and promotes myogenic commitment [45]. In contrast, DLK1 functions as a NOTCH antagonist, facilitating MuSC cell‐cycle exit during late differentiation to sustain the stem cell reservoir [46, 47, 48]. Our data demonstrate that high‐IMFF muscles are characterized by DLK1 downregulation and concomitant JAG1/NOTCH receptor activation in MuSCs, consistent with a niche state that destabilizes MuSC quiescence, promotes overactivation, and predisposes to exhaustion. Notably, the decline of DLK1 may also facilitate adipogenesis [47], creating a feed‐forward loop that exacerbates intramuscular fat accumulation. Parallel alterations were observed in growth factor signaling. MuSCs in high‐IMFF muscles received amplified BMP and FGF cues, consistent with earlier observations [17]. Excessive FGF2 disrupts MuSC quiescence and accelerates depletion [49], while BMP and FGF pathways promote proliferation but repress differentiation [50]. The concomitant upregulation of ACVR2A and FGFR1 in MuSCs further sensitizes them to these signals, with ACVR2A also mediating the catabolic effects of MSTN, thereby potentiating atrophy. Despite FAPs showing overall active proliferation and paracrine, their diminished secretion of IGF1, a principal factor for muscle regeneration, suggests an erosion of their regenerative potential [51]. Thus, the altered FAP secretome not only promotes overactivation but simultaneously deprives MuSCs of regenerative support. Together, these findings support a model in which intramuscular fat acts not merely as passive replacement of contractile tissue but as an active signaling hub that rewires the MuSC niche. In this model, the erosion of MuSC quiescence‐maintaining signals (e.g., DLK1) and the amplification of maladaptive paracrine inputs (e.g., JAG1/NOTCH, BMP5, FGF2) converge to destabilize quiescence, drive MuSC exhaustion, and impair regenerative capacity.

Previous studies have identified IMF as a pro‐inflammatory fat depot, similar to the visceral adipose tissue (VAT) [41]. The secretomes of IMF and VAT have been shown to promote inflammation, myotube atrophy, myotube lipid accumulation, and insulin resistance in vitro [52, 53]. However, in vivo evidence challenges this view: ectopic lipid deposition does not invariably cause muscle insulin resistance, as exemplified by young endurance athletes, a phenomenon referred to as the “athlete's paradox” [20]. More recently, studies have highlighted that the adipokine resistin selectively inhibits myogenesis in elderly myoblasts but not in younger cells, underscoring the pathological relevance of muscle fat infiltration in aging [23]. This study is the first to demonstrate that intramuscular adipocytes selectively inhibit the myogenic capacity of satellite cells through the ANGPTL4‐SDC2 axis in the spinal sarcopenia population, shedding light on the selective effects of adipokines on aged muscle. ANGPTL4, a secreted adipokine primarily expressed in adipose tissue and the liver, regulates circulating lipid metabolism through inhibition of LPL activity [54]. It also influences inflammation and vascular homeostasis by binding to integrins and/or NRP, exhibiting bidirectional effects [55]. Although one study has reported that ANGPTL4 inhibits myogenesis as a WNT antagonist, it remains unclear whether other ANGPTL4‐related signaling pathways, such as MAPK/ERK, Sonic Hedgehog, and cAMP/PKA, are involved in this process [30]. Our results demonstrated that ANGPTL4 treatment inhibited myotube formation in C2C12 cells, concomitant with downregulation of oxidative phosphorylation and FoxO signaling, and activation of AGE‐RAGE, JAK‐STAT, PI3K‐Akt, TNF, and chemokine pathways. SDC2 knockdown partially rescued ANGPTL4‐induced myogenic inhibition, concomitantly enhancing AMPK, TCA cycle, cGMP‐PKG, and fatty acid metabolism pathways, while suppressing TNF, IL‐17, and cellular senescence signaling (Figure S9A–D). These findings suggest that the ANGPTL4‐SDC2 axis inhibits myogenesis by disrupting metabolism, impairing energy supply, and promoting muscle inflammation and insulin resistance. Although ANGPTL4 is generally expressed in adipocytes, its selective inhibition of myogenesis depends on the expression of SDC2 in MuSCs. SDC2, a transmembrane heparan sulfate proteoglycan involved in cell adhesion and extracellular matrix interactions, is highly expressed in keloid tissues [56, 57]. SDC2 responds to regenerative signals such as TGFβ, FGF, and NOTCH [58, 59], and marks PAX7^+^CD54^+^ α9β1^+^myogenic progenitor cells, thus playing an active role in muscle regeneration [60]. We speculate that chronic inflammation, tissue remodeling, and enhanced muscle regeneration signals (e.g., BMPs, FGFs, HGFs, and PDGFs) in high‐IMFF muscles may activate the expression of SDC2 in MuSCs, potentially priming them for muscle regeneration (Figure 4F–K and Figure S6E–H). However, in the context of high IMFF, abundant adipose‐derived ANGPTL4 could overwhelm SDC2 in MuSCs, maneuvering the signaling toward inhibiting myogenesis. In return, abrupt regeneration could further activate quiescent MuSCs to compensate for the deficient muscle, thus forming a vicious cycle and eventually leading to muscle wasting and dysfunction. We speculate that this potential pathological positive feedback loop could underlie the paraspinal sarcopenia.

This study has several limitations. First, muscle samples were obtained from elderly patients with lumbar degenerative disease, and the influence of limited sample size and age‐related comorbidities cannot be excluded. Future studies including younger and middle‐aged individuals will be necessary to distinguish aging‐associated changes from features that are specifically related to pathological intramuscular fat infiltration. Second, as snRNA‐seq primarily captures nuclear transcripts, ligand‐receptor interactions inferred from transcriptomic analyses may not fully reflect actual protein secretion levels or ligand bioavailability, particularly for secreted factors such as ANGPTL4. To address this limitation, we complemented transcriptomic analyses with ELISA and functional assays, which supported the biological relevance of the ANGPTL4‐SDC2 signaling axis. Third, although our in vitro experiments demonstrated that ANGPTL4 inhibited C2C12 myogenic differentiation and that SDC2 knockdown partially rescued this phenotype, in vivo validation and confirmation across different skeletal muscles remain lacking. Rigorous evaluation of the physiological role of the ANGPTL4‐SDC2 signaling axis will require the establishment of an appropriate animal model that recapitulates the pathological characteristics of age‐related intramuscular fat infiltration, together with cell type‐specific genetic manipulation of ANGPTL4 and/or SDC2. These important questions will be addressed in our future studies. Finally, the absence of muscle single‐cell RNA sequencing data limited our ability to comprehensively characterize immune and inflammatory cell dynamics. Future studies integrating single‐cell and single‐nucleus transcriptomics with spatial profiling, imaging, and biopsy data from well‐characterized patient cohorts will provide a more comprehensive understanding of the cellular remodeling and pathological progression of intramuscular fat infiltration.

## 4 Experimental Section

### 4.1 Study Design

This study was designed to investigate the key pathogenic mechanisms underlying fat infiltration‐related spinal muscle atrophy. By integrating trunk muscle isokinetic strength assessments, MRI imaging analysis, advanced bulk and single‐nucleus transcriptomics, and validation using human muscle biopsies and primary cells, we identified an ANGPTL4–SDC2 axis within fat‐infiltrated muscles that influences MuSC myogenesis. Additionally, we provided a comprehensive characterization of fat‐infiltrated muscle composition, its microenvironment, and highly adipogenic FAP subpopulations, which were meticulously validated.

### 4.2 Participants and Ethics

This was a single‐center, cross‐sectional study conducted at the Spine Surgery Department of Beijing Jishuitan Hospital between June 2023 and June 2024. The study adhered to the principles of the Declaration of Helsinki and was approved by the Research Ethics Committee of Beijing Jishuitan Hospital, China (K2023‐138‐00). Written informed consent was obtained from all participants. Inclusion criteria were as follows: (I) age between 60 and 90 years; (II) main diagnosis of lumbar degenerative diseases; (III) Willingness to participate and signing of informed consent. The exclusion criteria were as follows: (I) body mass index (BMI) ≤18 kg/m^2^ or BMI ≥30 kg/m^2^; (II) significant pain during spinal movement; (III) an Oswestry Disability Index (ODI) score >80%; (IV) significant spinal deformity (congenital scoliosis with cobb angle >10° or lumbar spondylolisthesis higher than grade 1); (V) a history of lumbar trauma, lumbar surgery, or infectious diseases; (VI) a history of neurological, neuromuscular diseases, autoimmune diseases, tumors or dysphoria; (VII) use of medications known to affect muscle structure or function.

Participants were consecutively screened between June 2023 and June 2024. A total of 781 elderly Chinese patients were initially enrolled based on age and diagnosis. After applying the inclusion and exclusion criteria, 330 participants were eligible for further evaluation. The assessments included basic characteristics (age, sex, BMI), spinal functions (ODI and VAS back pain score), and general sarcopenia evaluations. Sarcopenia assessments followed standard protocols, including SARC‐F, Short Physical Performance Battery score (SPPB), gait speed, grip strength, and chair stand test. Dominant hand grip strengths were measured using a Jamar hand‐held dynamometer (Jamar, Los Angeles, CA) as reported in the literature [61, 62]. Possible sarcopenia was defined as low hand grip strength (<28 kg for males and <18 kg for females), and low physical performance (gait speed <1.0 m/s, SPPB ≤9, or chair stand test ≥12 s) according to the 2019 Consensus of Asian Working Group for Sarcopenia (AWGS 2019) [63]. Finally, 80 male and 81 female participants with complete imaging and strength data were included in the final analysis. The basic information of the 161 participants was summarized in Figure 2B. Complete metadata for the donors of samples used in bulk RNA‐seq, snRNA‐seq, and histological analysis is provided in Data file S1.

### 4.3 Acquisition of Imaging and Isokinetic Muscle Strength Data

As described in previous publications, all patients underwent standardized quantitative CT scans of the lumbar spine in the supine position. Magnetic resonance imaging (MRI) data of the lumbar spine were acquired using a 3.0 T scanner (Ingenia 3.0T, Philips, Netherlands). All CT scans were performed at 120 kVp with a slice thickness of 1 mm as reported in previous studies [64]. All MRI and CT scans were conducted with a standard lumbar scanning protocol by qualified and trained radiology technicians. After removing the patient information, the radiographic data were stored in DICOM format and exported on a dedicated hard disk. Axial scans of T2‐W1 MRI images and CT images at the L4/5 intervertebral disc level were selected for further analysis.

In CT images, the cross‐sectional trunk muscle area was segmented manually and measured within thresholds of ‐29 HU and 150 HU, as described in previous studies. For MRI images, the clustering‐based Otsu thresholding techniques were used to automatically distinguish fat and water signals from paraspinal muscles (including longissimus thoracis muscle, iliocostalis lumborum muscle, and multifidus muscles) as previously reported (Figure 1C) [65, 66]. Regions of interest (ROIs) were outlined manually. The total muscle cross‐sectional area (TCSA, cm^2^) was measured along muscle subfascial regions, while the muscle tissue area was measured as functional muscle cross‐sectional area (fCSA, cm^2^). Adipose tissues were measured in two categories: intramuscular fat (IMF, cm^2^, the fat within the muscle tissue boundary), and perimuscular fat (PMF, cm^2^, as the interpolation of total subfascial fat (TMF) minus intramuscular fat, PMF = TMF‐IMF). Corresponding proportions of intramuscular fat fraction (IMFF), perimuscular fat fraction (PMFF), and total muscle fat fraction (TMFF) were calculated as follows: IMFF = IMF /(fCSA+IMF) ×100%, PMFF = PMF /TCSA×100%, TMFF = TMF/TCSA×100%. The muscle mass was represented by the height‐adjusted CT/MRI muscle area, including paraspinal muscle index (PSMI, cm^2^/m^2^, PSMI = paraspinal muscle fCSA/height^2) from MRI and cross‐sectional muscle index (SMI, cm^2^/m^2^, SMI = cross‐sectional skeletal muscle area/height^2) from CT [64]. Two spine surgeons with 5 years of clinical and MRI reading experience assessed each image independently. Inter‐ and intra‐observer reliability were assessed using intraclass correlation coefficients (ICCs) (Table S3).

All participants underwent trunk muscle kinetic strength testing following a standard protocol. After a 5‐minute walk and 2‐min mobility measurements to warm up, participants performed rotation, flexion, extension, and lateral flexion isokinetic movements (with a 20‐s rest in between) at a speed of 15°/s in a standing position using the BioniX Sim3 Pro device (Physiomed, Germany) [67]. Each movement was repeated five times. Strength data were exported in PDF files. The trunk muscle strength performance was represented by the normalized trunk muscle peak strength (normalized strength = peak torque/body weight, Nm/kg). The normalized extension, rotation (average of left and right rotation), lateral flexion (average of left and right flexion), and average paraspinal strength were included in the final analysis.

### 4.4 Grouping, Data Cleaning, and Statistics Strategy

All data groupings and intergroup comparisons were conducted separately for females and males. Participants with incomplete data (e.g., missing MRI or strength measurements) and low‐quality data (e.g., poor image quality or incomplete strength data) were excluded during the quality control. Outliers exceeding mean ± 3 standard deviations (SD) were marked, remeasured, and retained if confirmed as valid. The normality of continuous variables was evaluated using the Kolmogorov‐Smirnov test, Q‐Q plots, and visual histograms (Table S4 and Figures S11 and S12). Normally distributed variables were reported as mean ± SD. Due to the skewed distribution of IMFF, correlation analyses involving IMFF were conducted using Spearman's correlation analysis. Differences in IMFF between females and males were assessed using the Mann‐Whitney *U* test (the test statistic is abbreviated as U hereinafter), while differences among IMFF groups were assessed using one‐way ANOVA with post‐hoc Tukey's test (Table S5). Univariate linear regression was performed to assess the influence of various muscle parameters on muscle mass and strength. Multiple linear regression was applied to identify factors influencing trunk muscle strength, with muscle strength as the dependent variable and age, sex, BMI, ODI, IMFF, and SMI as independent variables. Dancey and Reidy's classification was applied to interpret the strength of correlation coefficients: 0.1–0.3 was considered weak, 0.4–0.6 moderate, 0.7–0.9 strong, and 1 indicated a perfect correlation [68]. Restricted cubic spline (RCS) was used to model the association of IMFF, PMFF, and TMFF with standardized trunk muscle strengths (extension, rotation, and lateral flexion). Fully adjusted models were controlled for baseline sex, age, and the ODI. When a significant non‐linear association was detected (P < 0.05), segmented regression with a single inflection point was subsequently fitted. A log‐likelihood ratio test comparing a one‐line (non‐segmented) model to a segmented regression model was used to determine whether a threshold exists. The Mann–Whitney *U* test or two‐tailed unpaired t‐test was used to conduct comparisons of cell proportion and validation between two independent groups. All statistical analyses were conducted using R version 4.5.1 (R Foundation for Statistical Computing, Vienna, Austria), along with Zstats v1.0 (www.zstats.net). Statistical significance was defined as a two‐tailed *P* < 0.05.

### 4.5 Muscle Collection and Tissue Processing

Human multifidus muscle samples for bulk RNA‐seq, snRNA‐seq, and validation experiments were obtained with informed consent from patients. Multifidus muscle tissue was collected from the edge of the spinous process at the L4/5 intervertebral disc level during surgical procedures. After collection, the muscle tissue was immediately cleaned with wet gauze and trimmed to remove dead muscle, extramuscular fat, and excess tendon tissue from the muscle biopsies. The remaining muscle tissue was portioned (about 100 mg per sample), stored in cryopreservation tubes, and preserved in liquid nitrogen before processing. Additionally, one muscle sample was fixed in 4% paraformaldehyde for subsequent paraffin embedding and sectioning.

Seven‐micron paraffin sections were obtained from paraffin‐embedded samples using Leica microtomes (RM2016, Leica, Germany). Ten‐micron cryosections were obtained from OCT‐embedded frozen samples using a Thermo cryostat (CryoStar NX50, Thermo Fisher Scientific, USA).

### 4.6 Histology and Immunofluorescence

For H&E staining, slides were submerged in hematoxylin for 3 min followed by quick immersions in distilled water and tap water, then incubated in 95% ethanol for 1 min. Slides were next incubated in eosin for 15 sec, followed by quick immersions in 100% ethanol and xylene before Permount mounting. Brightfield images were taken using the NIKON ECLIPSE E100 microscope and DS‐U3 Digital Camera system (Nikon, Japan). Fourteen muscle samples were selected from the high and low IMFF groups (3 males and 4 females in each group). The slides were measured manually using Image J/FIJI software (National Institutes of Health; https://imagej.net/ij/). The minimum Feret diameters were measured to quantify myofiber size from at least 5 randomly chosen locations per sample, with a minimum of 50 fibers measured per location and at least 400 fibers measured per sample. The proportion of regenerating muscle fibers was calculated as the ratio of centrally nucleated fibers to the total number of fibers (% central nuclei = central nuclei fibers/total fibers × 100%).

For Picro Sirius Red (PSR) staining, slides were incubated in Picro Sirius Red A (G1078, Servicebio, China) for 2 min, followed by two washes and a 30‐min incubation in Picro Sirius Red B solution. The slides were then dehydrated in a graded ethanol series, cleared in xylene twice, and mounted with Permount. Brightfield images were captured as described previously. Ten muscle samples were selected for PSR staining, with 5 samples each from the high and low IMFF groups. For each muscle sample, 3 or 4 muscle tissue fields were randomly selected for collagen quantification. The collagen fraction was calculated using ImageJ software and was defined as the ratio of the stained collagen area to the total muscle tissue area, excluding blank regions.

For Oil Red O staining, fresh cryosections were fixed in 4% paraformaldehyde for 15 min at room temperature (RT). The sections were then stained with Oil Red O in 60% isopropanol for 10 min and counterstained with hematoxylin for 3 to 5 min. Stained sections were visualized under a light microscope. Sections of 7 low IMFF and 8 high IMFF samples were stained by Oil Red O and then measured using ImageJ software. The quantification of Oil Red O levels was expressed as the ratio of the stained lipid area within the muscle tissue to the total muscle tissue area, excluding blank regions.

For immunofluorescence, tissue sections were fixed in 4% paraformaldehyde (Servicebio, G1101 – 500 mL) for 30 min, permeabilized with 0.1% Triton X‐100 (Acmec, T88491) for 10 min, and blocked with 3% BSA (Solarbio, SW3015) in PBS for 30 min. Cells or tissue sections were incubated with the primary antibodies overnight at 4°C. Primary antibody dilutions were as follows: rabbit anti‐MYH7 (enriched in slow‐twitch type I muscle fibers, Servicebio, GB112131, 1:5000), rabbit anti‐MYH1 (enriched in fast‐twitch type IIx muscle fibers, Servicebio, GB112130, 1:5000), rabbit anti laminin (Sigma‐Aldrich, L9393, 1:200), mouse anti‐PAX7 (DSHB, PAX7, 1:50), rat anti‐PDGFRa (eBioscience, 17‐1401‐81, 1:100), rabbit anti‐VWF (Proteintech, 27186‐1‐AP, 1:100), rabbit anti peripilin (Cell Signaling Technology, 9349, 1:100), rabbit anti‐SDC2 (Thermo fisher, 36–6200, 1:100). Slides were then washed in 1xPBS 3 times, 5 min each and incubated with the appropriate secondary antibodies for 50 min at RT. Slides were washed again in PBS as above and mounted with DAPI solution (G1012, ServiceBio, China) and antifade mounting medium (G1401, ServiceBio, China) to visualize nuclei. Epifluorescent images were visualized using a Nikon Eclipse C1 microscope and scanned using a PANNORAMIC MIDI slide scanner (3DHISTECH, Sysmex, Switzerland).

### 4.7 Bulk RNA‐seq and Data Analysis

Total RNA was extracted from human multifidus muscles using the TRIzol method and sequenced on the NovaSeq platform. RNA‐seq data analysis was performed as previously described [69]. In brief, HISAT2 software (version 2.2.1) was used to align paired‐end RNA‐seq reads to the human transcriptome (GRCh38) [70]. Transcripts were assembled, and gene abundances were estimated using StringTie software (version 2.2.1) [71]. Weighted correlation network analysis was conducted using WGCNA (version 1.72.5) to calculate the association between gene networks and clinical indicators (Data file S2) [72].

### 4.8 10× Genomics 3’ Single‐Nucleus RNA‐seq

Skeletal muscle tissues were lysed using nuclei lysis buffer, and cell debris and large clumps were removed using a 40 µm Flowmi Cell Strainer. After myelin removal, single‐nucleus RNA‐seq libraries were constructed using 10× Genomics Chromium single cell 3’ reagent kit (v3.1) following standard protocols and sequenced on the NovaSeq platform (Data file S3).

### 4.9 snRNA‐seq Processing, Integration, Cell Clustering, and Annotation

Paired‐end snRNA‐seq reads were aligned to the human reference genome (GRCh38) using Cell Ranger (version 7.1.0). The count matrix of snRNA‐seq was filtered based on the criteria of 200< nFeature RNA < 7500 and 500< nCount RNA < 25000. Seurat (4.3.0) was used for global cell clustering [73]. The filtered matrix data were normalized using the NormalizeData function. The top 2000 highly variable genes were identified, and the data were scaled by regressing out nFeature_RNA and nCount_RNA. Dimensionality reduction was performed using principal component analysis. After batch effect correction using Harmony (1.2.0), cells were clustered using the RunUMAP function and annotated with canonical markers [74]. The FindAllMarkers function was used to identify marker genes for each cell cluster (Data file S4). Differentially expressed genes (DEGs) between low‐ and high‐fat infiltration groups were identified using the FindMarkers function, with a cutoff of adjusted p‐value < 0.05 and | log _2_ (fold change) | > 0.25. GO and KEGG functional enrichment analysis of DEGs was performed using ClusterProfiler (version 4.6.2) [75]. For MuSCs, adipocytes, ECs, FAPs, and myofibers re‐clustering, subsets were extracted from the global Seurat object and processed using the same methods described above (Data file S5).

### 4.10 Cell‐Cell Interaction Analysis

CellChat (version 1.6.1) was used to analyze receptor‐ligand interactions in the snRNA‐seq data of low or high‐fat infiltration groups, following the standard analytical protocol [76]. Briefly, the cell type information and gene expression matrix of low‐ or high‐fat infiltration groups were imported into CellChat. The computeCommunProb and computeCommunProbPathway functions were used to calculate the communication probability of receptor‐ligand interactions and signaling pathways (Data file S6). CellChat visualization tools were then used to compare the number and strength of the receptor‐ligand interactions and signaling pathways between the low‐ and high‐fat infiltration groups.

### 4.11 Pseudotime Analysis

For the adipocyte lineage tracing, the subclusters of adipocytes, FAP cells, endothelial cells, MuSC, and myofibers were selected for pseudotime analysis using Monocle 2 (version 2.24) [77]. Nuclei were ordered based on their gene expression profiles, and the top 2000 highly variable genes were selected for trajectory analysis. Differentially expressed genes in different branches of FAP‐EC and GPC3^+^FAP cells were identified.

### 4.12 Skeletal Muscle Biopsy Processing for Flow Cytometry and Cell Sorting

Freshly obtained multifidus muscle biopsies were minced with fine scissors and digested at 37°C for 60–90 min with gentle shaking in 10 mL (per gram of tissue) of solution containing 2.5 U/mL Dispase Il (Roche, 4942078001) and 1 mg/mL Collagenase II (Gibco, 17101015) supplemented with 5 mM MgCl_2_ (Solarbio, IM9034) and 2% penicillin‐streptavidin (Gibco, 15140122). Digestion was stopped with 10% FBS and 2 mM EDTA solution (Solarbio, E1170) in PBS, and the tissue suspension was sequentially filtered through 100‐µm (Falcon, 352360) and 40‐µm (Falcon, 352340) strainers to obtain the single‐cell suspensions. After centrifugation and reconstitution, cells were adjusted to 2 × 10^6^–7.5 × 10^6^ cells per milliliter with FACS buffer (2% FBS in PBS) and incubated with fluorophore‐antibody cocktails (Table S7) for 30 min to sort MuSCs (Lin^−^CD34‐CD82^+^CD56^+^), FAPs (Lin^−^CD34^+^CD56^−^), GPC3^+^FAPs (Lin^−^CD34^+^CD56^−^GPC3^+^), MME^+^FAPs (Lin^−^CD34^+^CD56^−^CD10^+^), and CD55^+^FAPs (Lin^−^CD34^+^CD56^−^CD55^+^). Cells were sorted and analyzed with an MA900 Multi‐Application Cell Sorter, and data were analyzed using FlowJo (version 10.4) software (Figure S10).

### 4.13 Cell Viability Assay

Sorted MuSCs were seeded in 96‐well plates at 1.0×10^4^ cells/well with a complete culture medium. After adherence for 24 h, the original medium was aspirated, and then cells were treated with different concentrations of recombinant human ANGPTL4 protein for 3 days. Cell viability was eventually determined by CCK‐8 (Solarbio, CA1210) assay according to the manufacturer's instructions (Beijing Solarbio Science &Technology Co., Ltd., China).

### 4.14 Cell Culture

To induce myogenic differentiation, C2C12 cells and flow‐sorted human MuSCs were harvested, and the cell concentration was adjusted to 100 000 cells/ml. The cells were seeded into 24‐well plates and cultured in complete culture medium (10% fetal bovine serum (Cytiva), 1% penicillin‐streptomycin (Gibco), DMEM/F‐12 medium (Gibco)). When the cell fusion degree reached over 80%, the medium was replaced with myogenic induction differentiation medium (2% Horse serum (Gibco), 1% Penicillin Streptomycin (Gibco), DMEM/F‐12 medium (Gibco)). In the ANGPTL4 treatment group, cells were cultured in differentiation medium containing 1 µg/mL recombinant human ANGPTL4. Control cells were cultured in differentiation medium without ANGPTL4. Both media were refreshed every 24 h. Upon reaching the 5th day of myoblast differentiation, the culture medium was carefully aspirated, and a single wash was carried out using PBS (Solarbio, P1020). The cells were then fixed, permeabilized, and blocked for immunofluorescence staining as previously described. Mature myotubes were stained with Myosin 4 monoclonal antibody (Invitrogen, 53‐6503‐82, 1:100) and Hoechst 33258 (Invitrogen, H3569). Myotube images were then collected using a fluorescence microscope (DMi8, Leica, Germany) and Leica Application Suite X 3.7.6.25997(LAS X, Leica, Germany), and the myotube diameter was calculated using ImageJ software.

To induce adipogenic differentiation, flow‐sorted FAP cells and their subsets were resuspended in complete medium; the concentration was adjusted to 2 × 10^5^ cells/ml, and the cells were spread in 12‐well plates. When the cell density reached 80%, the cells were replaced by lipid‐induced differentiation medium (OriCellTM, HUXXC‐90031). After 8 days, lipid droplet formation was detected by Nile Red staining (Solarbio, IN0170). Hoechst 33258 was used to stain nuclei (Thermo Fisher Scientific, Inc., 2683446). The stained cells were visualized using a fluorescence microscope (DMi8, Leica, Germany) and Leica Application Suite X 3.7.6.25997(LAS X, Leica, Germany), and the formation of lipid droplets was quantified using ImageJ software.

### 4.15 ELISA Assay of ANGPTL4

For ANGPTL4 protein detection in muscle samples, muscle samples with different degrees of fat infiltration were collected, weighed, and added with 1 mL RIPA lysate. After homogenization, the samples were centrifuged at 12000 rpm/min at 4°C for 10 min, and the supernatant was removed and stored at ‐80°C. For ANGPTL4 protein detection in the medium supernatant of FAP and its subsets, the medium supernatant was collected before (day 0) and after (day 8) adipogenic differentiation induction and stored at ‐80°C. ANGPTL4 protein levels were measured in tissue lysates and cell‐culture supernatants with the use of ELISA kits according to the manufacturer's instructions (Thermo, EHANGPTL4).

### 4.16 RNA Isolation and Quantitative Reverse Transcription PCR (qRT‐PCR)

According to the manufacturer's instructions (Spark Jade, AC0202‐B), total RNA was isolated from collected cells. The relative expression level of related genes adopted the standard ΔΔCt method. For C2C12 cells, Cyclophilin A was used as a loading control for normalization. For human cells, B2M was used as a loading control for normalization. Details of the primers are in Table S8.

### 4.17 Western Blot Assay

Western blot was used to detect the expression levels of MyoD (Santa Cruz, SC‐32758, dilution 1:1000), MyoG (DSHB, F5D, dilution 1:1000), and SDC2 (Thermo Fisher, 36–6200, dilution 1:250) proteins in muscle tissues or cells. Images were obtained through the Tanon 5800 automatic chemiluminescence image analyzer.

### 4.18 siRNA Transfection and ANGPTL4 Treatment

C2C12 cells were transfected with SDC2 siRNA (siSDC2‐#1, siSDC2‐#2, and negative control (siNC) with final concentration of 20 nmol/L GenePharma) using Lipofectamine RNAiMax (Invitrogen). After 24 and 72 h, the cells were harvested to assess knockdown efficiency using qRT‐PCR and Western blot, respectively. Then the three groups were treated with expansion medium, myogenic differentiation medium, or myogenic differentiation medium with 1 µg/ml ANGPTL4 for 5 days. Subsequently, myosin 4 staining and Western blot analysis were performed. The siRNA sequences used were as follows:

siNC (negative control): 5’‐UUCUCCGAACGUGUCACGU‐3’;

siSDC2‐#1: 5’‐UUCAGGAGUAUAUCCUAUU‐3’;

siSDC2‐#2: 5’‐CGGAGAAACAUUCAGACAA‐3’.

## Author Contributions

Conceptualization: SBB, DLA, MPW YL, LW, WW, RW, DW. Methodology: DW, RW, QR, WW, JL. Validation: DW, QR, WL, YW, YZ. Formal analysis: DW, WW, RW. Investigation: DW, WW, QR, WL, YW, YZ. Resources: WW, GY, RY, CL, ZZ, YL. Data curation: WW, DW, WZ, YY, ZC, LW, XC. Interpretation of data and statistical analysis: WW, FD, QR, DW. Writing – drafting of the manuscript: WW, DW, QR. Writing – review & editing: RW, LW, FD, XC, JL, YL. Visualization: WW, DW, QR. Supervision: RW, LW, YL. Project administration: RW. Funding acquisition: YL, LW, WW, XC

## Funding

This work was supported by Beijing Municipal Health Commission (grant number BJRITO‐RDP‐2025). The Youth Beijing Scholars Program (grant number NO.025). Talent program of Ministry of Science and Technology (grant number No.SQ2022RA2A000031). Beijing Postdoctoral Research Foundation (grant number No.2023‐zz‐030) and Beijing Hospitals Authority Ascent Plan (grant number DFL20220401)

## Conflicts of Interest

The authors declare no conflicts of interest.

## Supporting information




**Supporting File 1**: advs77648‐sup‐0001‐SuppMat.docx.

**Supporting File 2**: advs77648‐sup‐0002‐SuppMat.zip.

## Data Availability

The datasets generated in this study are available on the NCBI BioProject Archive under accession no. PRJNA1193134. Correspondence and requests for materials should be addressed to Prof. Yajun Liu.
