# Supplementary material for: Single‐Nucleus Atlas of Spinal Sarcopenia Patients Reveals Disruption of Quiescent and Regenerative Satellite Cell Niches in Fatty‐Infiltrated Muscle
Source: Adv Sci (Weinh). 2026 Oct 4:e77648. Online ahead of print. doi: 10.1002/advs.77648 (PMC13634468; doi:10.1002/advs.77648)
Supplement: Supplementary file 1 [file ADVS-9999-e77648-s001.docx]

**Supplementary Information (Supplementary Table S1-S7, Supplementary Figure S1-S12)**

Table S1.1. Results of multiple linear regression analysis for the impact of IMFF on trunk muscle strengths

Table S1.2. Results of multiple linear regression analysis for the impact of PMFF on trunk muscle strengths

Table S2.1. Threshold effect analysis of IMFF on trunk extension strength

Table S2.2. Threshold effect analysis of PMFF on trunk extension strength

Table S2.3. Threshold effect analysis of TMFF on trunk extension strength

Table S3. The inter- and intra-observer agreement over MRI measurements using the intraclass correlation coefficient (ICC)

Table S4. Tests of normality in males and females

Table S5. One-way ANOVA and post-hoc Tukey's multiple comparison analyses of muscle mass, strength and sarcopenia indicators among IMFF groups

Table S6. One-way ANOVA and post-hoc Tukey's multiple comparison analyses of Nile red staining among primary FAP subtype adipogenesis

Table S7. Detailed information of fluorescent dye-conjugated antibodies used in flow cytometry

Table S8. Primers sequences used in this study

Figure S1. Correlation between IMFF, PMFF, and general sarcopenia indicators

Figure S2. Nonlinear associations between muscle fat infiltration and strength, and group comparisons based on IMFF

Figure S3. Quality control of the snRNA-seq of human multifidus muscles

Figure S4. Single-nucleus transcriptomic atlas of fat infiltration in human multifidus muscle

Figure S5. The microenvironment of fat infiltration in human multifidus muscle

Figure S6. Intercellular communication networks in human multifidus muscle with low and high fat infiltration

Figure S7. Intercellular interaction networks of ANGPTL signaling pathway across sex and cell subtypes

Figure S8. Pseudo-trajectory analysis of MuSC, FAPs, ECs, and adipocytes

Figure S9. KEGG enrichment analysis of differentially expressed genes associated with AGNPTL4 co-culture and siSDC2 treatment

Figure S10. Flow cytometric analysis of human MuSCs and FAPs

Figure S11. Q-Q plots (A) and visual histograms (B) of continuous variables in males

Figure S12. Q-Q plots (A) and visual histograms (B) of continuous variables in females

## **Supplementary Table S1.1. Results of multiple linear regression analysis for the impact of IMFF on trunk muscle strengths**

| **Models** | **variables** | **Unstandardized Coefficients** | | **Standardized Coefficients** | **t** | **P-value** | **95.0% confidence interval** | | **Collinearity Statistics** | |
| --- | --- | --- | --- | --- | --- | --- | --- | --- | --- | --- |
|  |  | **B** | **Std. Error** | **Beta** |  |  | **Lower** | **Upper** | **Tolerance** | **VIF** |
| **Extension** | **Constant** | 1.723 | 0.517 |  | 3.336 | 0.001 | 0.703 | 2.743 |  |  |
|  | **Age** | -0.012 | 0.005 | -0.175 | -2.267 | 0.025 | -0.023 | -0.002 | 0.874 | 1.145 |
|  | **Sex** | 0.069 | 0.069 | 0.097 | 0.994 | 0.322 | -0.068 | 0.205 | 0.548 | 1.825 |
|  | **BMI** | -0.016 | 0.011 | -0.123 | -1.518 | 0.131 | -0.038 | 0.005 | 0.799 | 1.252 |
|  | **ODI** | -0.388 | 0.171 | -0.175 | -2.268 | 0.025 | -0.726 | -0.05 | 0.883 | 1.132 |
|  | **SMI** | 0.011 | 0.006 | 0.208 | 1.941 | 0.054 | 0 | 0.022 | 0.457 | 2.19 |
|  | **IMFF** | -0.809 | 0.368 | -0.177 | -2.2 | 0.029 | -1.536 | -0.083 | 0.811 | 1.233 |
| **Flexion** | **Constant** | 1.973 | 0.474 |  | 4.163 | 0 | 1.037 | 2.91 |  |  |
|  | **Age** | -0.012 | 0.005 | -0.187 | -2.498 | 0.014 | -0.022 | -0.003 | 0.874 | 1.145 |
|  | **Sex** | -0.062 | 0.063 | -0.093 | -0.984 | 0.327 | -0.188 | 0.063 | 0.548 | 1.825 |
|  | **BMI** | -0.003 | 0.01 | -0.022 | -0.285 | 0.776 | -0.022 | 0.017 | 0.799 | 1.252 |
|  | **ODI** | -0.615 | 0.157 | -0.291 | -3.914 | 0 | -0.925 | -0.305 | 0.883 | 1.132 |
|  | **SMI** | 0.003 | 0.005 | 0.063 | 0.606 | 0.545 | -0.007 | 0.013 | 0.457 | 2.19 |
|  | **IMFF** | -0.626 | 0.337 | -0.144 | -1.855 | 0.066 | -1.292 | 0.041 | 0.811 | 1.233 |
| **Rotation** | **Constant** | 0.464 | 0.171 |  | 2.707 | 0.008 | 0.125 | 0.803 |  |  |
|  | **Age** | -0.003 | 0.002 | -0.125 | -1.555 | 0.122 | -0.006 | 0.001 | 0.874 | 1.145 |
|  | **Sex** | 0.018 | 0.023 | 0.081 | 0.803 | 0.423 | -0.027 | 0.064 | 0.548 | 1.825 |
|  | **BMI** | -0.001 | 0.004 | -0.018 | -0.209 | 0.834 | -0.008 | 0.006 | 0.799 | 1.252 |
|  | **ODI** | -0.069 | 0.057 | -0.097 | -1.222 | 0.224 | -0.182 | 0.043 | 0.883 | 1.132 |
|  | **SMI** | 0.003 | 0.002 | 0.159 | 1.439 | 0.152 | -0.001 | 0.006 | 0.457 | 2.19 |
|  | **IMFF** | -0.312 | 0.122 | -0.213 | -2.556 | **0.012** | -0.553 | -0.071 | 0.811 | 1.233 |
| **Lateral Flexion** | **Constant** | 0.792 | 0.313 |  | 2.529 | 0.012 | 0.174 | 1.411 |  |  |
|  | **Age** | -0.004 | 0.003 | -0.099 | -1.282 | 0.202 | -0.011 | 0.002 | 0.874 | 1.145 |
|  | **Sex** | 0.049 | 0.042 | 0.113 | 1.163 | 0.247 | -0.034 | 0.131 | 0.548 | 1.825 |
|  | **BMI** | -0.006 | 0.007 | -0.07 | -0.866 | 0.388 | -0.019 | 0.007 | 0.799 | 1.252 |
|  | **ODI** | -0.296 | 0.104 | -0.219 | -2.855 | 0.005 | -0.501 | -0.091 | 0.883 | 1.132 |
|  | **SMI** | 0.009 | 0.003 | 0.272 | 2.546 | 0.012 | 0.002 | 0.016 | 0.457 | 2.19 |
|  | **IMFF** | -0.448 | 0.223 | -0.161 | -2.007 | **0.046** | -0.888 | -0.007 | 0.811 | 1.233 |
| **Average** | **Constant** | 0.996 | 0.294 |  | 3.388 | 0.001 | 0.415 | 1.576 |  |  |
|  | **Age** | -0.006 | 0.003 | -0.155 | -2.042 | 0.043 | -0.012 | 0 | 0.874 | 1.145 |
|  | **Sex** | 0.042 | 0.039 | 0.103 | 1.074 | 0.284 | -0.035 | 0.12 | 0.548 | 1.825 |
|  | **BMI** | -0.008 | 0.006 | -0.1 | -1.255 | 0.211 | -0.02 | 0.004 | 0.799 | 1.252 |
|  | **ODI** | -0.255 | 0.097 | -0.198 | -2.622 | 0.01 | -0.448 | -0.063 | 0.883 | 1.132 |
|  | **SMI** | 0.007 | 0.003 | 0.241 | 2.29 | 0.023 | 0.001 | 0.014 | 0.457 | 2.19 |
|  | **IMFF** | -0.523 | 0.209 | -0.197 | -2.502 | **0.013** | -0.937 | -0.11 | 0.811 | 1.233 |

Note: the impact of age, sex, BMI (body mass index), ODI (Oswestry Disability Index), and SMI (skeletal muscle index) were adjusted.

## **Supplementary Table S1.2. Results of multiple linear regression analysis for the impact of PMFF on trunk muscle strengths**

| **Models** | **variables** | **Unstandardized Coefficients** | | **Standardized Coefficients** | **t** | **P-value** | **95.0% confidence interval** | | **Collinearity Statistics** | |
| --- | --- | --- | --- | --- | --- | --- | --- | --- | --- | --- |
|  |  | **B** | **Std. Error** | **Beta** |  |  | **Lower** | **Upper** | **Tolerance** | **VIF** |
| **Extension** | **Constant** | 1.859 | 0.525 |  | 3.542 | 0.001 | 0.822 | 2.896 |  |  |
|  | **Age** | -0.014 | 0.005 | -0.204 | -2.625 | 0.01 | -0.025 | -0.003 | 0.893 | 1.12 |
|  | **Sex** | 0.069 | 0.07 | 0.097 | 0.978 | 0.33 | -0.07 | 0.207 | 0.547 | 1.827 |
|  | **BMI** | -0.02 | 0.011 | -0.153 | -1.882 | 0.062 | -0.042 | 0.001 | 0.812 | 1.232 |
|  | **ODI** | -0.423 | 0.173 | -0.19 | -2.444 | 0.016 | -0.765 | -0.081 | 0.889 | 1.125 |
|  | **SMI** | 0.013 | 0.006 | 0.248 | 2.26 | 0.025 | 0.002 | 0.025 | 0.448 | 2.234 |
|  | **PMFF** | -0.257 | 0.467 | -0.044 | -0.551 | 0.583 | -1.179 | 0.665 | 0.833 | 1.2 |
| **Flexion** | **Constant** | 2.103 | 0.479 |  | 4.394 | 0 | 1.158 | 3.049 |  |  |
|  | **Age** | -0.013 | 0.005 | -0.205 | -2.749 | 0.007 | -0.023 | -0.004 | 0.893 | 1.12 |
|  | **Sex** | -0.064 | 0.064 | -0.095 | -0.994 | 0.322 | -0.19 | 0.063 | 0.547 | 1.827 |
|  | **BMI** | -0.005 | 0.01 | -0.041 | -0.518 | 0.605 | -0.025 | 0.014 | 0.812 | 1.232 |
|  | **ODI** | -0.636 | 0.158 | -0.301 | -4.026 | 0 | -0.948 | -0.324 | 0.889 | 1.125 |
|  | **SMI** | 0.004 | 0.005 | 0.081 | 0.774 | 0.44 | -0.006 | 0.015 | 0.448 | 2.234 |
|  | **PMFF** | -0.402 | 0.425 | -0.073 | -0.945 | 0.346 | -1.242 | 0.438 | 0.833 | 1.2 |
| **Rotation** | **Constant** | 0.532 | 0.174 |  | 3.056 | 0.003 | 0.188 | 0.876 |  |  |
|  | **Age** | -0.003 | 0.002 | -0.15 | -1.861 | 0.065 | -0.007 | 0 | 0.893 | 1.12 |
|  | **Sex** | 0.018 | 0.023 | 0.078 | 0.76 | 0.448 | -0.028 | 0.064 | 0.547 | 1.827 |
|  | **BMI** | -0.002 | 0.004 | -0.042 | -0.498 | 0.619 | -0.009 | 0.005 | 0.812 | 1.232 |
|  | **ODI** | -0.079 | 0.057 | -0.111 | -1.376 | 0.171 | -0.192 | 0.034 | 0.889 | 1.125 |
|  | **SMI** | 0.003 | 0.002 | 0.182 | 1.602 | 0.111 | -0.001 | 0.007 | 0.448 | 2.234 |
|  | **PMFF** | -0.227 | 0.155 | -0.122 | -1.467 | 0.145 | -0.533 | 0.079 | 0.833 | 1.2 |
| **Lateral Flexion** | **Constant** | 0.879 | 0.317 |  | 2.771 | 0.006 | 0.252 | 1.505 |  |  |
|  | **Age** | -0.005 | 0.003 | -0.121 | -1.572 | 0.118 | -0.011 | 0.001 | 0.893 | 1.12 |
|  | **Sex** | 0.048 | 0.042 | 0.112 | 1.137 | 0.257 | -0.035 | 0.132 | 0.547 | 1.827 |
|  | **BMI** | -0.008 | 0.007 | -0.093 | -1.149 | 0.252 | -0.021 | 0.005 | 0.812 | 1.232 |
|  | **ODI** | -0.313 | 0.105 | -0.231 | -2.991 | 0.003 | -0.519 | -0.106 | 0.889 | 1.125 |
|  | **SMI** | 0.01 | 0.004 | 0.298 | 2.739 | 0.007 | 0.003 | 0.017 | 0.448 | 2.234 |
|  | **PMFF** | -0.234 | 0.282 | -0.066 | -0.831 | 0.407 | -0.791 | 0.322 | 0.833 | 1.2 |
| **Average** | **Constant** | 1.092 | 0.299 |  | 3.645 | 0 | 0.5 | 1.683 |  |  |
|  | **Age** | -0.007 | 0.003 | -0.184 | -2.409 | 0.017 | -0.013 | -0.001 | 0.893 | 1.12 |
|  | **Sex** | 0.042 | 0.04 | 0.102 | 1.045 | 0.298 | -0.037 | 0.121 | 0.547 | 1.827 |
|  | **BMI** | -0.01 | 0.006 | -0.13 | -1.624 | 0.107 | -0.022 | 0.002 | 0.812 | 1.232 |
|  | **ODI** | -0.276 | 0.099 | -0.214 | -2.793 | 0.006 | -0.471 | -0.081 | 0.889 | 1.125 |
|  | **SMI** | 0.009 | 0.003 | 0.278 | 2.575 | 0.011 | 0.002 | 0.015 | 0.448 | 2.234 |
|  | **PMFF** | -0.233 | 0.266 | -0.069 | -0.874 | 0.384 | -0.758 | 0.293 | 0.833 | 1.2 |

Note: the impact of age, sex, BMI (body mass index), ODI (Oswestry Disability Index), and SMI (skeletal muscle index) were adjusted.

## **Supplementary Table S2.1. Threshold effect analysis of IMFF on trunk extension strength**

| Outcome | β (95%CI) | *P* |
| --- | --- | --- |
| Model 1 Fitting model by standard linear regression | -1.02 (-1.72 - -0.32) | 0.005 |
| Model 2 Fitting model by two-piecewise linear regression |  |  |
| Infection point | 0.07 |  |
| <0.07 | -7.54 (-12.69 - -2.39) | 0.006 |
| ≥0.07 | -0.19 (-1.01 - -0.62) | 0.641 |
| P for likelihood test |  | <0.001 |

## **Supplementary Table S2.2. Threshold effect analysis of PMFF on trunk extension strength**

| Outcome | β (95%CI) | *P* |
| --- | --- | --- |
| Model 1 Fitting model by standard linear regression | -0.58 (-1.47 - -0.31) | 0.201 |
| Model 2 Fitting model by two-piecewise linear regression |  |  |
| Infection point | 0.32 |  |
| <0.32 | -0.97 (-2.02 - -0.09) | 0.075 |
| ≥0.32 | 19.15 (12.96 - 25.34) | 0.004 |
| P for likelihood test |  | 0.031 |

## **Supplementary Table S2.3. Threshold effect analysis of TMFF on trunk extension strength**

| Outcome | β (95%CI) | *P* |
| --- | --- | --- |
| Model 1 Fitting model by standard linear regression | -0.72 (-1.28 - -0.17) | 0.012 |
| Model 2 Fitting model by two-piecewise linear regression |  |  |
| Infection point | 0.23 |  |
| <0.23 | -2.88 (-5.78 - -0.01) | 0.058 |
| ≥0.23 | -0.20 (-0.94 - 0.54) | 0.600 |
| P for likelihood test |  | 0.034 |

## **Supplementary Table S3. The inter- and intra-observer agreement over MRI measurements using the intraclass correlation coefficient (ICC)**

| **Parameter** | **Intra-observer** | **95% CI** | **P** | **Inter-observer** | **95% CI** | **P** |
| --- | --- | --- | --- | --- | --- | --- |
| **TCSA** | 0.982 | (0.934, 0.993) | <0.001 | 0.952 | (0.911, 0.974) | <0.001 |
| **TFA** | 0.978 | (0.956, 0.988) | <0.001 | 0.966 | (0.751, 0.989) | <0.001 |
| **MULMA** | 0.965 | (0.919, 0.983) | <0.001 | 0.896 | (0.771, 0.949) | <0.001 |
| **ESMA** | 0.978 | (0.883, 0.992) | <0.001 | 0.944 | (0.897, 0.970) | <0.001 |
| **MULTF** | 0.967 | (0.939, 0.983) | <0.001 | 0.954 | (0.915, 0.975) | <0.001 |
| **MULIF** | 0.902 | (0.823, 0.947) | <0.001 | 0.827 | (0.695, 0.904) | <0.001 |
| **ESIF** | 0.987 | (0.975, 0.993) | <0.001 | 0.974 | (0.951, 0.986) | <0.001 |

Abbr. TCSA: total cross-sectional area, TFA: total fat area, MULMA: multifidus muscle area, ESMA: erector spinae muscle area, MULTF: multifidus muscle total fat area, MULIF: multifidus muscle intramuscular fat area, ESIF: erector spinae muscle intramuscular fat area. CI: confidence interval.

## **Supplementary Table S4. Tests of normality in males and females**

|  | **Male** | | | | | | **Female** | | | | | |
| --- | --- | --- | --- | --- | --- | --- | --- | --- | --- | --- | --- | --- |
|  | **Kolmogorov-Smirnov a** | | | **Shapiro-Wilk** | | | **Kolmogorov-Smirnov a** | | | **Shapiro-Wilk** | | |
|  | **Statistic** | **df** | **Sig.** | **Statistic** | **df** | **Sig.** | **Statistic** | **df** | **Sig.** | **Statistic** | **df** | **Sig.** |
| **Age** | 0.116 | 80 | 0.009 | 0.968 | 80 | 0.04 | 0.112 | 81 | 0.014 | 0.969 | 81 | 0.044 |
| **BMI** | 0.074 | 80 | .200* | 0.973 | 80 | 0.086 | 0.083 | 81 | .200* | 0.973 | 81 | 0.087 |
| **IMFF** | 0.132 | 80 | 0.002 | 0.878 | 80 | 0 | 0.171 | 81 | 0 | 0.853 | 81 | 0 |
| **PMFF** | 0.065 | 80 | .200* | 0.982 | 80 | 0.311 | 0.082 | 81 | .200* | 0.977 | 81 | 0.144 |
| **TFF** | 0.056 | 80 | .200* | 0.977 | 80 | 0.156 | 0.142 | 81 | 0 | 0.954 | 81 | 0.005 |
| **ODI** | 0.09 | 80 | 0.167 | 0.977 | 80 | 0.164 | 0.091 | 81 | 0.091 | 0.979 | 81 | 0.202 |
| **VAS** | 0.136 | 80 | 0.001 | 0.957 | 80 | 0.008 | 0.13 | 81 | 0.002 | 0.962 | 81 | 0.016 |
| **GS** | 0.085 | 80 | .200* | 0.969 | 80 | 0.052 | 0.09 | 81 | 0.156 | 0.984 | 81 | 0.395 |
| **ST** | 0.178 | 80 | 0 | 0.833 | 80 | 0 | 0.156 | 81 | 0 | 0.879 | 81 | 0 |
| **Gait Speed** | 0.066 | 80 | .200* | 0.985 | 80 | 0.492 | 0.124 | 81 | 0.004 | 0.965 | 81 | 0.028 |
| **SMI** | 0.081 | 80 | .200* | 0.964 | 80 | 0.025 | 0.065 | 81 | .200* | 0.964 | 81 | 0.023 |
| **Mult_index** | 0.093 | 80 | 0.085 | 0.974 | 80 | 0.105 | 0.082 | 81 | .200* | 0.983 | 81 | 0.374 |
| **Erec_index** | 0.078 | 80 | .200* | 0.987 | 80 | 0.578 | 0.091 | 81 | 0.093 | 0.968 | 81 | 0.043 |
| **PSMI** | 0.056 | 80 | .200* | 0.989 | 80 | 0.763 | 0.068 | 81 | .200* | 0.986 | 81 | 0.521 |
| **E** | 0.06 | 80 | .200* | 0.976 | 80 | 0.135 | 0.135 | 81 | 0.001 | 0.924 | 81 | 0 |
| **F** | 0.067 | 80 | .200* | 0.989 | 80 | 0.762 | 0.075 | 81 | .200* | 0.975 | 81 | 0.11 |
| **ROT** | 0.089 | 80 | 0.179 | 0.969 | 80 | 0.048 | 0.096 | 81 | 0.064 | 0.973 | 81 | 0.081 |
| **LF** | 0.07 | 80 | .200* | 0.986 | 80 | 0.561 | 0.098 | 81 | 0.052 | 0.976 | 81 | 0.135 |
| **Ave** | 0.056 | 80 | .200* | 0.986 | 80 | 0.528 | 0.109 | 81 | 0.019 | 0.966 | 81 | 0.03 |

* This is a lower bound of the true significance.

a. Liliefors Significance Correction.

## **Supplementary Table S5. One-way ANOVA and post-hoc Tukey's multiple comparison analyses of muscle mass, strength and sarcopenia indicators among IMFF groups**

|  | **IMFF Groups** | | | |  |  |
| --- | --- | --- | --- | --- | --- | --- |
| **Male** | **<5%** | **5-10%** | **10-15%** | **>15%** | ***F*** | ***P*** |
| **Mult_index** | 561.8±92.6 | 497.1±109.0 | 479.3±83.0 | 355.6±142.5 | 10.242 | 0 |
| **Erec_index** | 914.0±203.2 | 834.3±187.1 | 732.4±208.1 | 677.9±183.1 | 5.051 | 0.003 |
| **PSMI** | 1475.9±231.5 | 1331.4±241.2 | 1211.7±264.5 | 1033.5±229.1 | 9.834 | 0 |
| **SMI** | 46.1±5.7 | 43.4±4.8 | 41.7±5.1 | 40.2±5.2 | 4.177 | 0.009 |
| **E** | 1.0±0.3 | 0.7±0.3 | 0.7±0.3 | 0.6±0.3 | 6.526 | 0.001 |
| **ROT** | 0.4±0.1 | 0.3±0.1 | 0.4±0.1 | 0.2±0.1 | 4.996 | 0.003 |
| **LF** | 0.7±0.2 | 0.7±0.2 | 0.6±0.2 | 0.4±0.2 | 7.017 | 0 |
| **Ave** | 0.7±0.2 | 0.6±0.2 | 0.6±0.2 | 0.4±0.2 | 7.485 | 0 |
| **GS** | 34.7±8.1 | 32.0±7.3 | 31.7±8.4 | 32.7±8.3 | 0.623 | 0.602 |
| **Speed** | 1.1±0.3 | 0.9±0.2 | 1.0±0.3 | 1.0±0.2 | 1.286 | 0.285 |
| **ST** | 11.5±3.0 | 16.1±6.9 | 14.1±3.7 | 14.6±3.4 | 3.7 | 0.015 |
| **Female** | **<5%** | **5-10%** | **10-15%** | **>15%** | **F** | **P** |
| **Mult_index** | 454.0±64.8 | 442.1±83.42 | 422.2±75.7 | 347.2±93.8 | 6.547 | 0.001 |
| **Erec_index** | 856.4±225.4 | 742.6±133.6 | 733.3±179.1 | 652.5±176.2 | 3.34 | 0.024 |
| **PSMI** | 1310.3±178.9 | 1184.7±186.7 | 1155.5±181.1 | 999.7±196.6 | 7.279 | 0 |
| **SMI** | 33.5±2.8 | 35.5±5.2 | 36.4±5.9 | 33.6±4.3 | 1.487 | 0.225 |
| **E** | 1.1±0.5 | 0.7±0.3 | 0.7±0.3 | 0.6±0.3 | 6.352 | 0.001 |
| **ROT** | 0.4±0.1 | 0.3±0.1 | 0.3±0.1 | 0.3±0.1 | 3.716 | 0.015 |
| **LF** | 0.7±0.2 | 0.6±0.2 | 0.6±0.2 | 0.5±0.2 | 2.373 | 0.077 |
| **Ave** | 0.8±0.3 | 0.5±0.2 | 0.5±0.2 | 0.5±0.2 | 5.585 | 0.002 |
| **GS** | 22.6±3.6 | 20.8±4.7 | 21.8±5.8 | 20.1±5.9 | 0.663 | 0.577 |
| **Speed** | 1.0±0.4 | 1.0±0.3 | 1.0±0.3 | 0.9±0.2 | 0.719 | 0.543 |
| **ST** | 15.6±3.5 | 17.3±7.0 | 15.7±4.6 | 15.9±4.3 | 0.514 | 0.674 |

| **Tukey’s multiple comparison analyses for males** | | | | | | | |
| --- | --- | --- | --- | --- | --- | --- | --- |
| **Dependent Variable** | **(I) IMFF_Level** | **(J) IMFF_Level** | **Mean Difference (I-J)** | **Std. Error** | **Sig.** | **95% Confidence Interval** | |
|  |  |  |  |  |  | Lower Bound | Upper Bound |
| **Mult_index** | 1 | 2 | 64.7447675600* | 29.80 | 0.03 | 5.40 | 124.09 |
|  |  | 3 | 82.5105844000* | 33.05 | 0.02 | 16.69 | 148.33 |
|  |  | 4 | 206.2324502000* | 37.40 | 0.00 | 131.75 | 280.71 |
|  | 2 | 1 | -64.7447675600* | 29.80 | 0.03 | -124.09 | -5.40 |
|  |  | 3 | 17.77 | 31.95 | 0.58 | -45.88 | 81.41 |
|  |  | 4 | 141.4876827000* | 36.43 | 0.00 | 68.92 | 214.05 |
|  | 3 | 1 | -82.5105844000* | 33.05 | 0.02 | -148.33 | -16.69 |
|  |  | 2 | -17.77 | 31.95 | 0.58 | -81.41 | 45.88 |
|  |  | 4 | 123.7218658000* | 39.14 | 0.00 | 45.78 | 201.67 |
|  | 4 | 1 | -206.2324502000* | 37.40 | 0.00 | -280.71 | -131.75 |
|  |  | 2 | -141.4876827000* | 36.43 | 0.00 | -214.05 | -68.92 |
|  |  | 3 | -123.7218658000* | 39.14 | 0.00 | -201.67 | -45.78 |
| **Erec_index** | 1 | 2 | 79.72 | 55.65 | 0.16 | -31.11 | 190.56 |
|  |  | 3 | 181.6689403000* | 61.72 | 0.00 | 58.75 | 304.59 |
|  |  | 4 | 236.1609887000* | 69.84 | 0.00 | 97.07 | 375.25 |
|  | 2 | 1 | -79.72 | 55.65 | 0.16 | -190.56 | 31.11 |
|  |  | 3 | 101.94 | 59.68 | 0.09 | -16.91 | 220.80 |
|  |  | 4 | 156.4363254000* | 68.04 | 0.02 | 20.92 | 291.95 |
|  | 3 | 1 | -181.6689403000* | 61.72 | 0.00 | -304.59 | -58.75 |
|  |  | 2 | -101.94 | 59.68 | 0.09 | -220.80 | 16.91 |
|  |  | 4 | 54.49 | 73.09 | 0.46 | -91.07 | 200.06 |
|  | 4 | 1 | -236.1609887000* | 69.84 | 0.00 | -375.25 | -97.07 |
|  |  | 2 | -156.4363254000* | 68.04 | 0.02 | -291.95 | -20.92 |
|  |  | 3 | -54.49 | 73.09 | 0.46 | -200.06 | 91.07 |
| **PSMI** | 1 | 2 | 144.4694309000* | 68.73 | 0.04 | 7.59 | 281.35 |
|  |  | 3 | 264.1795247000* | 76.22 | 0.00 | 112.37 | 415.99 |
|  |  | 4 | 442.3934389000* | 86.25 | 0.00 | 270.61 | 614.18 |
|  | 2 | 1 | -144.4694309000* | 68.73 | 0.04 | -281.35 | -7.59 |
|  |  | 3 | 119.71 | 73.70 | 0.11 | -27.08 | 266.50 |
|  |  | 4 | 297.9240080000* | 84.03 | 0.00 | 130.56 | 465.29 |
|  | 3 | 1 | -264.1795247000* | 76.22 | 0.00 | -415.99 | -112.37 |
|  |  | 2 | -119.71 | 73.70 | 0.11 | -266.50 | 27.08 |
|  |  | 4 | 178.21 | 90.27 | 0.05 | -1.56 | 357.99 |
|  | 4 | 1 | -442.3934389000* | 86.25 | 0.00 | -614.18 | -270.61 |
|  |  | 2 | -297.9240080000* | 84.03 | 0.00 | -465.29 | -130.56 |
|  |  | 3 | -178.21 | 90.27 | 0.05 | -357.99 | 1.56 |
| **SMI** | 1 | 2 | 2.68 | 1.48 | 0.07 | -0.26 | 5.62 |
|  |  | 3 | 4.36832570300* | 1.64 | 0.01 | 1.11 | 7.63 |
|  |  | 4 | 5.87863322500* | 1.85 | 0.00 | 2.19 | 9.57 |
|  | 2 | 1 | -2.68 | 1.48 | 0.07 | -5.62 | 0.26 |
|  |  | 3 | 1.69 | 1.58 | 0.29 | -1.46 | 4.84 |
|  |  | 4 | 3.20 | 1.80 | 0.08 | -0.39 | 6.79 |
|  | 3 | 1 | -4.36832570300* | 1.64 | 0.01 | -7.63 | -1.11 |
|  |  | 2 | -1.69 | 1.58 | 0.29 | -4.84 | 1.46 |
|  |  | 4 | 1.51 | 1.94 | 0.44 | -2.35 | 5.37 |
|  | 4 | 1 | -5.87863322500* | 1.85 | 0.00 | -9.57 | -2.19 |
|  |  | 2 | -3.20 | 1.80 | 0.08 | -6.79 | 0.39 |
|  |  | 3 | -1.51 | 1.94 | 0.44 | -5.37 | 2.35 |
| **Ext** | 1 | 2 | .289386031000* | 0.09 | 0.00 | 0.12 | 0.46 |
|  |  | 3 | .313184560000* | 0.10 | 0.00 | 0.12 | 0.50 |
|  |  | 4 | .404143250000* | 0.11 | 0.00 | 0.19 | 0.62 |
|  | 2 | 1 | -.289386031000* | 0.09 | 0.00 | -0.46 | -0.12 |
|  |  | 3 | 0.02 | 0.09 | 0.80 | -0.16 | 0.21 |
|  |  | 4 | 0.11 | 0.11 | 0.28 | -0.09 | 0.32 |
|  | 3 | 1 | -.313184560000* | 0.10 | 0.00 | -0.50 | -0.12 |
|  |  | 2 | -0.02 | 0.09 | 0.80 | -0.21 | 0.16 |
|  |  | 4 | 0.09 | 0.11 | 0.42 | -0.13 | 0.32 |
|  | 4 | 1 | -.404143250000* | 0.11 | 0.00 | -0.62 | -0.19 |
|  |  | 2 | -0.11 | 0.11 | 0.28 | -0.32 | 0.09 |
|  |  | 3 | -0.09 | 0.11 | 0.42 | -0.32 | 0.13 |
| **ROT** | 1 | 2 | 0.06 | 0.03 | 0.06 | 0.00 | 0.13 |
|  |  | 3 | 0.03 | 0.04 | 0.38 | -0.04 | 0.10 |
|  |  | 4 | .153683115000* | 0.04 | 0.00 | 0.07 | 0.23 |
|  | 2 | 1 | -0.06 | 0.03 | 0.06 | -0.13 | 0.00 |
|  |  | 3 | -0.03 | 0.03 | 0.39 | -0.10 | 0.04 |
|  |  | 4 | .091360886700* | 0.04 | 0.02 | 0.01 | 0.17 |
|  | 3 | 1 | -0.03 | 0.04 | 0.38 | -0.10 | 0.04 |
|  |  | 2 | 0.03 | 0.03 | 0.39 | -0.04 | 0.10 |
|  |  | 4 | .121562761000* | 0.04 | 0.01 | 0.04 | 0.21 |
|  | 4 | 1 | -.153683115000* | 0.04 | 0.00 | -0.23 | -0.07 |
|  |  | 2 | -.091360886700* | 0.04 | 0.02 | -0.17 | -0.01 |
|  |  | 3 | -.121562761000* | 0.04 | 0.01 | -0.21 | -0.04 |
| **LF** | 1 | 2 | 0.08 | 0.05 | 0.13 | -0.03 | 0.19 |
|  |  | 3 | .157626805000* | 0.06 | 0.01 | 0.04 | 0.28 |
|  |  | 4 | .302929725000* | 0.07 | 0.00 | 0.17 | 0.44 |
|  | 2 | 1 | -0.08 | 0.05 | 0.13 | -0.19 | 0.03 |
|  |  | 3 | 0.07 | 0.06 | 0.21 | -0.04 | 0.19 |
|  |  | 4 | .219445029000* | 0.07 | 0.00 | 0.09 | 0.35 |
|  | 3 | 1 | -.157626805000* | 0.06 | 0.01 | -0.28 | -0.04 |
|  |  | 2 | -0.07 | 0.06 | 0.21 | -0.19 | 0.04 |
|  |  | 4 | .145302920000* | 0.07 | 0.05 | 0.00 | 0.29 |
|  | 4 | 1 | -.302929725000* | 0.07 | 0.00 | -0.44 | -0.17 |
|  |  | 2 | -.219445029000* | 0.07 | 0.00 | -0.35 | -0.09 |
|  |  | 3 | -.145302920000* | 0.07 | 0.05 | -0.29 | 0.00 |
| **Ave** | 1 | 2 | .145064318000* | 0.05 | 0.01 | 0.04 | 0.25 |
|  |  | 3 | .167643906000* | 0.06 | 0.00 | 0.06 | 0.28 |
|  |  | 4 | .286918697000* | 0.06 | 0.00 | 0.16 | 0.41 |
|  | 2 | 1 | -.145064318000* | 0.05 | 0.01 | -0.25 | -0.04 |
|  |  | 3 | 0.02 | 0.05 | 0.68 | -0.09 | 0.13 |
|  |  | 4 | .141854378000* | 0.06 | 0.03 | 0.02 | 0.27 |
|  | 3 | 1 | -.167643906000* | 0.06 | 0.00 | -0.28 | -0.06 |
|  |  | 2 | -0.02 | 0.05 | 0.68 | -0.13 | 0.09 |
|  |  | 4 | 0.12 | 0.07 | 0.08 | -0.01 | 0.25 |
|  | 4 | 1 | -.286918697000* | 0.06 | 0.00 | -0.41 | -0.16 |
|  |  | 2 | -.141854378000* | 0.06 | 0.03 | -0.27 | -0.02 |
|  |  | 3 | -0.12 | 0.07 | 0.08 | -0.25 | 0.01 |

| **Tukey’s multiple comparison analyses for females** | | | | | | | |
| --- | --- | --- | --- | --- | --- | --- | --- |
| **Dependent Variable** | **(I) IMFF_Level** | **(J) IMFF_Level** | **Mean Difference (I-J)** | **Std. Error** | **Sig.** | **95% Confidence Interval** | |
|  |  |  |  |  |  | Lower Bound | Upper Bound |
| **Mult_index** | 1 | 2 | 11.90 | 30.32 | 0.70 | -48.48 | 72.28 |
|  |  | 3 | 31.76 | 31.39 | 0.32 | -30.75 | 94.26 |
|  |  | 4 | 106.7189603000* | 31.62 | 0.00 | 43.75 | 169.69 |
|  | 2 | 1 | -11.90 | 30.32 | 0.70 | -72.28 | 48.48 |
|  |  | 3 | 19.85 | 23.45 | 0.40 | -26.84 | 66.55 |
|  |  | 4 | 94.8172388600* | 23.76 | 0.00 | 47.50 | 142.13 |
|  | 3 | 1 | -31.76 | 31.39 | 0.32 | -94.26 | 30.75 |
|  |  | 2 | -19.85 | 23.45 | 0.40 | -66.55 | 26.84 |
|  |  | 4 | 74.9633896900* | 25.11 | 0.00 | 24.96 | 124.97 |
|  | 4 | 1 | -106.7189603000* | 31.62 | 0.00 | -169.69 | -43.75 |
|  |  | 2 | -94.8172388600* | 23.76 | 0.00 | -142.13 | -47.50 |
|  |  | 3 | -74.9633896900* | 25.11 | 0.00 | -124.97 | -24.96 |
| **Erec_index** | 1 | 2 | 113.73 | 62.74 | 0.07 | -11.20 | 238.67 |
|  |  | 3 | 123.04 | 64.95 | 0.06 | -6.30 | 252.38 |
|  |  | 4 | 203.9135399000* | 65.43 | 0.00 | 73.62 | 334.21 |
|  | 2 | 1 | -113.73 | 62.74 | 0.07 | -238.67 | 11.20 |
|  |  | 3 | 9.31 | 48.52 | 0.85 | -87.31 | 105.92 |
|  |  | 4 | 90.18 | 49.16 | 0.07 | -7.72 | 188.08 |
|  | 3 | 1 | -123.04 | 64.95 | 0.06 | -252.38 | 6.30 |
|  |  | 2 | -9.31 | 48.52 | 0.85 | -105.92 | 87.31 |
|  |  | 4 | 80.87 | 51.96 | 0.12 | -22.59 | 184.33 |
|  | 4 | 1 | -203.9135399000* | 65.43 | 0.00 | -334.21 | -73.62 |
|  |  | 2 | -90.18 | 49.16 | 0.07 | -188.08 | 7.72 |
|  |  | 3 | -80.87 | 51.96 | 0.12 | -184.33 | 22.59 |
| **PSMI** | 1 | 2 | 125.64 | 68.87 | 0.07 | -11.50 | 262.77 |
|  |  | 3 | 154.7969379000* | 71.30 | 0.03 | 12.82 | 296.77 |
|  |  | 4 | 310.6325001000* | 71.83 | 0.00 | 167.61 | 453.66 |
|  | 2 | 1 | -125.64 | 68.87 | 0.07 | -262.77 | 11.50 |
|  |  | 3 | 29.16 | 53.26 | 0.59 | -76.90 | 135.22 |
|  |  | 4 | 184.9963444000* | 53.97 | 0.00 | 77.53 | 292.46 |
|  | 3 | 1 | -154.7969379000* | 71.30 | 0.03 | -296.77 | -12.82 |
|  |  | 2 | -29.16 | 53.26 | 0.59 | -135.22 | 76.90 |
|  |  | 4 | 155.8355622000* | 57.03 | 0.01 | 42.27 | 269.40 |
|  | 4 | 1 | -310.6325001000* | 71.83 | 0.00 | -453.66 | -167.61 |
|  |  | 2 | -184.9963444000* | 53.97 | 0.00 | -292.46 | -77.53 |
|  |  | 3 | -155.8355622000* | 57.03 | 0.01 | -269.40 | -42.27 |
| **SMI** | 1 | 2 | -1.96 | 1.83 | 0.29 | -5.59 | 1.67 |
|  |  | 3 | -2.86 | 1.89 | 0.13 | -6.63 | 0.90 |
|  |  | 4 | -0.12 | 1.90 | 0.95 | -3.91 | 3.67 |
|  | 2 | 1 | 1.96 | 1.83 | 0.29 | -1.67 | 5.59 |
|  |  | 3 | -0.90 | 1.41 | 0.52 | -3.72 | 1.91 |
|  |  | 4 | 1.84 | 1.43 | 0.20 | -1.01 | 4.69 |
|  | 3 | 1 | 2.86 | 1.89 | 0.13 | -0.90 | 6.63 |
|  |  | 2 | 0.90 | 1.41 | 0.52 | -1.91 | 3.72 |
|  |  | 4 | 2.75 | 1.51 | 0.07 | -0.26 | 5.76 |
|  | 4 | 1 | 0.12 | 1.90 | 0.95 | -3.67 | 3.91 |
|  |  | 2 | -1.84 | 1.43 | 0.20 | -4.69 | 1.01 |
|  |  | 3 | -2.75 | 1.51 | 0.07 | -5.76 | 0.26 |
| **Ext** | 1 | 2 | .416843211000* | 0.13 | 0.00 | 0.17 | 0.67 |
|  |  | 3 | .436881687000* | 0.13 | 0.00 | 0.18 | 0.69 |
|  |  | 4 | .564588314000* | 0.13 | 0.00 | 0.30 | 0.82 |
|  | 2 | 1 | -.416843211000* | 0.13 | 0.00 | -0.67 | -0.17 |
|  |  | 3 | 0.02 | 0.10 | 0.84 | -0.17 | 0.21 |
|  |  | 4 | 0.15 | 0.10 | 0.14 | -0.05 | 0.34 |
|  | 3 | 1 | -.436881687000* | 0.13 | 0.00 | -0.69 | -0.18 |
|  |  | 2 | -0.02 | 0.10 | 0.84 | -0.21 | 0.17 |
|  |  | 4 | 0.13 | 0.10 | 0.22 | -0.08 | 0.33 |
|  | 4 | 1 | -.564588314000* | 0.13 | 0.00 | -0.82 | -0.30 |
|  |  | 2 | -0.15 | 0.10 | 0.14 | -0.34 | 0.05 |
|  |  | 3 | -0.13 | 0.10 | 0.22 | -0.33 | 0.08 |
| **ROT** | 1 | 2 | .080641558800* | 0.04 | 0.03 | 0.01 | 0.15 |
|  |  | 3 | .084986896200* | 0.04 | 0.03 | 0.01 | 0.16 |
|  |  | 4 | .126356331000* | 0.04 | 0.00 | 0.05 | 0.20 |
|  | 2 | 1 | -.080641558800* | 0.04 | 0.03 | -0.15 | -0.01 |
|  |  | 3 | 0.00 | 0.03 | 0.88 | -0.05 | 0.06 |
|  |  | 4 | 0.05 | 0.03 | 0.11 | -0.01 | 0.10 |
|  | 3 | 1 | -.084986896200* | 0.04 | 0.03 | -0.16 | -0.01 |
|  |  | 2 | 0.00 | 0.03 | 0.88 | -0.06 | 0.05 |
|  |  | 4 | 0.04 | 0.03 | 0.17 | -0.02 | 0.10 |
|  | 4 | 1 | -.126356331000* | 0.04 | 0.00 | -0.20 | -0.05 |
|  |  | 2 | -0.05 | 0.03 | 0.11 | -0.10 | 0.01 |
|  |  | 3 | -0.04 | 0.03 | 0.17 | -0.10 | 0.02 |
| **LF** | 1 | 2 | .181951413000* | 0.08 | 0.02 | 0.03 | 0.34 |
|  |  | 3 | .185924233000* | 0.08 | 0.02 | 0.03 | 0.34 |
|  |  | 4 | .196783798000* | 0.08 | 0.02 | 0.04 | 0.36 |
|  | 2 | 1 | -.181951413000* | 0.08 | 0.02 | -0.34 | -0.03 |
|  |  | 3 | 0.00 | 0.06 | 0.95 | -0.11 | 0.12 |
|  |  | 4 | 0.01 | 0.06 | 0.81 | -0.11 | 0.13 |
|  | 3 | 1 | -.185924233000* | 0.08 | 0.02 | -0.34 | -0.03 |
|  |  | 2 | 0.00 | 0.06 | 0.95 | -0.12 | 0.11 |
|  |  | 4 | 0.01 | 0.06 | 0.87 | -0.12 | 0.14 |
|  | 4 | 1 | -.196783798000* | 0.08 | 0.02 | -0.36 | -0.04 |
|  |  | 2 | -0.01 | 0.06 | 0.81 | -0.13 | 0.11 |
|  |  | 3 | -0.01 | 0.06 | 0.87 | -0.14 | 0.12 |
| **Ave** | 1 | 2 | .231359680000* | 0.07 | 0.00 | 0.09 | 0.37 |
|  |  | 3 | .235930939000* | 0.07 | 0.00 | 0.09 | 0.38 |
|  |  | 4 | .299084084000* | 0.07 | 0.00 | 0.15 | 0.45 |
|  | 2 | 1 | -.231359680000* | 0.07 | 0.00 | -0.37 | -0.09 |
|  |  | 3 | 0.00 | 0.05 | 0.93 | -0.10 | 0.11 |
|  |  | 4 | 0.07 | 0.06 | 0.23 | -0.04 | 0.18 |
|  | 3 | 1 | -.235930939000* | 0.07 | 0.00 | -0.38 | -0.09 |
|  |  | 2 | 0.00 | 0.05 | 0.93 | -0.11 | 0.10 |
|  |  | 4 | 0.06 | 0.06 | 0.29 | -0.05 | 0.18 |
|  | 4 | 1 | -.299084084000* | 0.07 | 0.00 | -0.45 | -0.15 |
|  |  | 2 | -0.07 | 0.06 | 0.23 | -0.18 | 0.04 |
|  |  | 3 | -0.06 | 0.06 | 0.29 | -0.18 | 0.05 |

Note: IMFF_Level：1: <5%, 2: 5-10%, 3: 10-15%, 4: >15%

## **Supplementary Table S6. One-way ANOVA and post-hoc Tukey's multiple comparison analyses of Nile red staining among primary FAP subtype adipogenesis**

| **ANOVA** | **Sum of Squares** | **df** | **Mean Square** | ***F* value** | ***P* value** |
| --- | --- | --- | --- | --- | --- |
| **Between Groups** | 5.85 | 3 | 1.95 | 6.976 | 0.006 |
| **Within Groups** | 3.354 | 12 | 0.279 |  |  |
| **Total** | 9.204 | 15 |  |  |  |

| **Tukey’s multiple comparison Dependent Variable: Nile_red_fold** | | | | | | |
| --- | --- | --- | --- | --- | --- | --- |
| **(I) Groups** | **(J) Groups** | **Mean Difference (I-J)** | **Std. Error** | **Sig.** | **95% Confidence Interval** | |
|  |  |  |  |  | **Lower Bound** | **Upper Bound** |
| FAP | GPC3^+^FAP | -1.11445* | 0.37383 | 0.049 | -2.2243 | -0.0046 |
|  | MME^+^FAP | 0.10441 | 0.37383 | 0.992 | -1.0054 | 1.2143 |
|  | CD55^+^FAP | 0.51933 | 0.37383 | 0.529 | -0.5905 | 1.6292 |
| GPC3^+^FAP | FAP | 1.11445* | 0.37383 | 0.049 | 0.0046 | 2.2243 |
|  | MME^+^FAP | 1.21886* | 0.37383 | 0.03 | 0.109 | 2.3287 |
|  | CD55^+^FAP | 1.63377* | 0.37383 | 0.004 | 0.5239 | 2.7436 |
| MME^+^FAP | FAP | -0.10441 | 0.37383 | 0.992 | -1.2143 | 1.0054 |
|  | GPC3^+^FAP | -1.21886* | 0.37383 | 0.03 | -2.3287 | -0.109 |
|  | CD55^+^FAP | 0.41491 | 0.37383 | 0.691 | -0.6949 | 1.5248 |
| CD55^+^FAP | FAP | -0.51933 | 0.37383 | 0.529 | -1.6292 | 0.5905 |
|  | GPC3^+^FAP | -1.63377* | 0.37383 | 0.004 | -2.7436 | -0.5239 |
|  | MME^+^FAP | -0.41491 | 0.37383 | 0.691 | -1.5248 | 0.6949 |

## **Supplementary Table S7. Detailed information of fluorescent dye-conjugated antibodies used in flow cytometry**

| **Antibodies** | **Fluorescent dye** | **Manufacturer** | **Cat. No.** |
| --- | --- | --- | --- |
| APC/Cyanine7 anti-human CD31 | APC-eFluor™ 780 | Biolegend | 303120 |
| CD45 Monoclonal Antibody (2D1) | APC-eFluor™ 780 | Thermo Fisher | 47-9459-42 |
| CD34 Monoclonal Antibody (4H11) | PE-Cyanine7 | Thermo Fisher | 25-0349-42 |
| anti-human CD82 Antibody | PerCP/Cyanine5.5 | Biolegend | 342112 |
| CD56 (NCAM) Monoclonal Antibody (CMSSB) | PE | Thermo Fisher | 12-0567-42 |
| Glypican 3 Recombinant Rabbit Monoclonal Antibody | APC | Thermo Fisher | MA5-40986 |
| CD55 Recombinant Rabbit Monoclonal Antibody | PERCP | Thermo Fisher | MA5-40928 |
| CD10 Monoclonal Antibody | Alexa Fluor™ 700 | Thermo Fisher | 56-0106-42 |

## **Supplementary Table S8. Primers sequences used in this study**

| **Genes** | **Sequences (5'-3')** |
| --- | --- |
| Homo_SDC2-F | TTGACAACAGCTCCATTGAAGAA |
| Homo_SDC2-R | CAGCTCTGGACTCTCTACATCC |
| Mus_SDC2-F | ACAAGGAGGAAGTTGACAT |
| Mus_SDC2-R | ACCAATAGCAGGATGAGG |
| Mus_Myod1-F | CCACTCCGGGACATAGACTTG |
| Mus_Myod1-R | AAAAGCGCAGGTCTGGTGAG |
| Homo_MME-F | AGAAGAAACAGCGATGGACTCC |
| Homo_MME-R | CATAGAGTGCGATCATTGTCACA |
| Homo_CD55-F | AGGCCGTACAAGTTTTCCCG |
| Homo_CD55-R | CCTTCTCGCCAGGAATTTTCAC |
| Homo_GPC3-F | CCTTTGAAATTGTTGTTCGCCA |
| Homo_GPC3-R | CCTGGGTTCATTAGCTGGGTA |
| Mus_Cyclophilin A-F | AAGAAGGCATGAACATTGTGGAAGC |
| Mus_Cyclophilin A-R | CGGAAATGGTGATCTTCTTGCTGG |

**Supplementary Figure S1. Correlation between IMFF, PMFF, and general sarcopenia indicators**

A). Scatterplot and correlation analysis of IMFF and PMFF in males and females. B). Scatter plots and simple linear regression of intramuscular and perimuscular fat in patients of different ages. C-G). Scatter plots with linear regression analyses showing associations of IMFF and PMFF with grip strength, stand test, gait speed, short physical performance battery (SPPB), and SARC-F questionnaire score (SARCF). H). Heatmap showing Spearmen correlation coefficients between general sarcopenia parameters, trunk muscle strengths, and paraspinal muscle characteristics. ^*^: *P*<0.05, ^**^: *P*<0.01.

**Supplementary Figure S2. Nonlinear associations between muscle fat infiltration and strength, and group comparisons based on IMFF**

A, B). Restricted cubic spline (RCS) curves and corresponding inflection points illustrating the nonlinear effects of IMFF, PMFF, and TMFF on standardized trunk lateral flexion (A) and rotation (B) strength. C-E). Comparisons of muscle mass indices (PSMI, SMI, multifidus and erector spinae muscle index) and strength parameters (normalized rotation and mean paraspinal strength) across IMFF groups in males and females. F). Distribution of IMFF values and prevalence of possible sarcopenia in males and females.

**Supplementary Figure S3. Quality control of the snRNA-seq of human multifidus muscles**

A). Bar plot showing the number of nuclei detected in each sample. B). Combined bar and line plot showing the reads (Log_10_ ratio) for each nucleus and the sequencing saturation in each sample, respectively. C, D). Violin plots showing the number of detected genes (nFeature_RNA; C) and total counts (nCount_RNA, D) in each sample after filtering out low-quality nuclei. E-G). Comparisons of muscle characteristics between low and high IMFF groups included in snRNA-seq, including muscle fat infiltration (multifidus and total paraspinal muscle IMFF), muscle mass (PSMI and SMI), and muscle strength (normalized trunk extension, rotation, lateral flexion, and mean trunk strength).

**Supplementary Figure S4. Single-nucleus transcriptomic atlas of fat infiltration in human multifidus muscle**

A). Dot plot showing the expression level of representative marker genes across each cell type. The size and color of the dots represent the percentage of cells expressing the marker genes and the average gene expression level, respectively. B). UMAP plots showing 112,154 snRNA-seq profiles grouped by low and high IMFF. C). Stacked bar chart displaying the cell proportion of low and high IMFF groups. D). UMAP plots showing snRNA-seq profiles grouped by sex. E). Stacked bar chart displaying the cell type composition in male and female groups. F). Stacked bar chart displaying the proportion of each cell type in each sample. G). Stacked bar chart displaying the comtribution of each sample in each cell type. H). Bar plot illustrating the number of nuclei for each cell type. I). Box plot showing the proportion of each cell type for each sample. Triangles and circles represent male and female samples, respectively. J, K). Representative immunofluorescence images showing intramuscular endothelial cells (red) and adipocytes (green) in low and high IMFF multifidus muscles.

**Supplementary Figure S5. Cellular microenvironment of fat infiltration in human multifidus muscle**

A-C) Dot plot showing representative marker gene expression in subpopulations of MuSCs (A), adipocytes (B), and FAPs (C). Dot size indicates the proportion of cells expressing each gene, and color represents the average expression level. D) UMAP plots of endothelial cells (ECs) identifying six major EC subpopulations in human multifidus muscle. E, F) Dot plots showing representative marker gene expression across EC (E) and MFI (F) subpopulations. G) UMAP plots of MFII nuclei revealing six distinct subpopulations in human multifidus muscle. H) Dot plot showing representative marker gene expression across MFII subpopulations.

**Supplementary Figure S6. Intercellular communication networks in human multifidus muscle with low and high fat infiltration**

A-J). Predicted intercellular signaling networks of CD46 (A), JAM (C), PDGF (E), HGF (G), and COLLAGEN (I) pathways in low and high IMFF groups. Corresponding dot plots (B, D, F, H, J) display representative ligand-receptor gene expression across cell types. Dot size represents the proportion of expressing cells, and color indicates the average expression level.

**Supplementary Figure S7. Intercellular interaction networks of ANGPTL signaling pathway across sex and cell subtypes**

A, B) Predicted ANGPTL signaling network in low and high IMFF groups in male (A) and female (B) samples. C, D) ANGPTL signaling networks and representative ligand-receptor gene expression patterns among MuSC, FAP, and adipocyte subtypes in low and high IMFF groups.

**Supplementary Figure S8. Pseudo-trajectory analysis of MuSCs, FAPs, ECs, and adipocytes**

A, B) Pseudo-trajectory analysis identifying a single differentiation trajectory in MuSCs and adipocytes. C, D) Distribution of MuSC and adipocyte subpopulations along the inferred trajectories. E, F) Pseudo-trajectory analysis revealing five distinct states among MF-I, MF-II, MuSC, and adipocyte populations. G, H) Distribution of MF-I, MF-II, and subpopulations of MuSCs and adipocytes along trajectories. I) Distribution of FAP and adipocyte subpopulations along trajectories. J, K) Pseudo-trajectory analysis identifying three differentiation states in ECs and adipocytes. L, M) Distribution of EC and adipocyte subpopulations along trajectories. N) Distribution of FAP-EC populations along cell trajectories in low and high IMFF groups.

**Supplementary Figure S9. KEGG pathway enrichment analysis of differentially expressed genes following AGNPTL4 co-culture and siSDC2 treatment**

A, B) KEGG enrichment analysis of down- and up-regulated genes in C2C12 cells co-cultured with recombinant ANGPTL4 protein compared with the control cells cultured without ANGPTL4. C, D) KEGG enrichment analysis of down- and up-regulated genes in C2C12 cells pretreated with siSDC2-#2 and co-cultured with ANGPTL4, compared with cells pretreated with negative control siRNA (siNC) and co-cultured with ANGPTL4.

**Supplementary Figure S10.** **Flow cytometric analysis of human MuSCs and FAPs**

A, B) Graded analysis of human MuSCs, FAPs, and FAP subpopulations in multifidus muscle single-cell suspension through flow cytometry. C-F) Flow cytometry validation of the purity of isolated FAPs (C), GPC3^+^ FAPs (D), MME^+^ FAPs (E), and CD55^+^ FAPs (F).

**Supplementary Figure S11. Q-Q plots (A) and visual histograms (B) of continuous variables in males**

**Supplementary Figure S12. Q-Q plots (A) and visual histograms (B) of continuous variables in females**

Data files list:

Data file 1 Donor metadata

Data file 2 WCGNA

Data file 3 Library information

Data file 4 markers of main cell groups

Data file 5 markers of cell subpopulations

Data file 6 cellchat

Please download these data by following the link:

<https://1drv.ms/f/c/1aa4dd02006f3c3c/IgBpxxOB5xbXTqLiq3TaM5EMARzajkSfG1mwuTsWG9ycxl8?e=s7HPXf>
